# Co-incidence of Damage and Microbial Patterns Controls Localized Immune Responses in Roots

**DOI:** 10.1016/j.cell.2020.01.013

**Published:** 2020-02-06

**Authors:** Feng Zhou, Aurélia Emonet, Valérie Dénervaud Tendon, Peter Marhavy, Dousheng Wu, Thomas Lahaye, Niko Geldner

**Affiliations:** 1 Department of Plant Molecular Biology, Biophore, UNIL-Sorge, University of Lausanne, 1015 Lausanne, Switzerland; 2 ZMBP-General Genetics, University of Tübingen, Auf der Morgenstelle 32, 72076 Tübingen, Germany

**Keywords:** *Arabidopsis*, root immunity, microbe patterns, pattern-recognition receptors, localized response, damage-gating

## Abstract

Recognition of microbe-associated molecular patterns (MAMPs) is crucial for the plant’s immune response. How this sophisticated perception system can be usefully deployed in roots, continuously exposed to microbes, remains a mystery. By analyzing MAMP receptor expression and response at cellular resolution in *Arabidopsis,* we observed that differentiated outer cell layers show low expression of pattern-recognition receptors (PRRs) and lack MAMP responsiveness. Yet, these cells can be gated to become responsive by neighbor cell damage. Laser ablation of small cell clusters strongly upregulates *PRR* expression in their vicinity, and elevated receptor expression is sufficient to induce responsiveness in non-responsive cells. Finally, localized damage also leads to immune responses to otherwise non-immunogenic, beneficial bacteria. Damage-gating is overridden by receptor overexpression, which antagonizes colonization. Our findings that cellular damage can “switch on” local immune responses helps to conceptualize how MAMP perception can be used despite the presence of microbial patterns in the soil.

## Highlights

• *Arabidopsis* roots request cell damage to mount a strong, localized immune response
• Damaged cells upregulate pattern-recognition receptor expression in their neighbors
• Endodermal barriers compartmentalize immune responses in differentiated cell-types
• Damage-gating can minimize immune responses against non-pathogenic root colonizers

## Graphical Abstract

**Figure:**
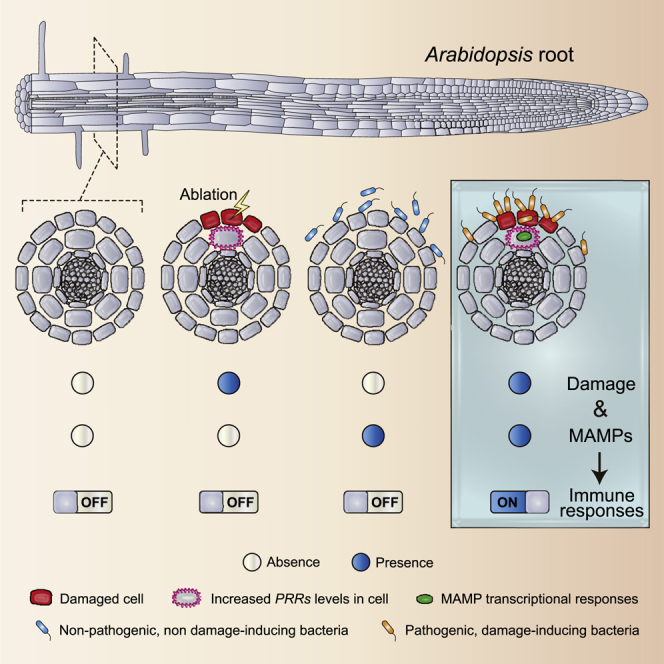

## Teaser

Roots require both microbial molecular patterns and plant tissue damage in order to mount localized antibacterial immune responses, revealing an effective way to respond appropriately to pathogens while sparing commensals.

## Introduction

A number of defined molecular patterns and corresponding receptors have been identified and shown to elicit a conserved set of molecular responses (Macho and Zipfel, 2014). However, identical microbial patterns from symbiotic or commensal microbes should be equally perceived (Pel and Pieterse, 2013). This is especially apparent in the microbe-rich soil environment of roots, whose outer cell layers do not possess protective barriers comparable to leaves. Recent breakthroughs in root microbiome research have heightened the interest in understanding how constitutive activation of PRRs by non-pathogenic microbes is avoided, while maintaining their effectiveness in defense (Castrillo et al., 2017, Finkel et al., 2017, Garrido-Oter et al., 2018, Yu et al., 2019). The molecular outlines of microbe-associated molecular pattern (MAMP) perception were characterized in systems allowing for quantitative, time-resolved measurements of early responses (Felix et al., 1999). In *Arabidopsis* (Chinchilla et al., 2006, Gómez-Gómez et al., 1999), leaf-disk reactive oxygen species (ROS) assays, phosphorylated mitogen-activated protein kinase (MAPK) blots, quantitative PCR (qPCR), or genome-wide transcription profiling became popular tools (Zipfel et al., 2004, Zipfel et al., 2006). Although such assays establish the molecular components of PRR signal transduction, they do not allow for a meaningful degree of spatial resolution, because they average cellular responses across entire organs. Actual, initial pathogen/microbe contacts, however, are localized to a few cells and cell types and this highly relevant spatial dimension of responses has remained largely unresolved. When studied, significant differences between single-cell and whole seedling responses were observed (Thor and Peiter, 2014). Roots mount an autonomous MAMP response (Poncini et al., 2017, Wyrsch et al., 2015) and β-glucuronidase (GUS) reporters, or callose deposition, revealed a restricted response to high concentrations of the bacterial MAMP, flg22, mainly in the root cap and root transition/elongation zone (Jacobs et al., 2011, Millet et al., 2010). GUS reporter assays are destructive, however, and remain below single-cell or tissue resolution. Moreover, the causes of this spatially restricted MAMP response have remained obscure, as well as its potential biological relevance.

In order to address these questions, we combined new and recently published fluorescent marker lines, based on a triple mVENUS fused to a nuclear localization signal (NLS-3xmVENUS) (Poncini et al., 2017, Vermeer et al., 2014). This allows for analysis of MAMP responses *in vivo* and at true cellular resolution. These highly sensitive markers were selected for good expression and stable responses, across transgenic lines and in successive generations. The promoters selected were based on well-established and widely used MAMP responsive genes. *PER5* (*PEROXIDASE 5*) was chosen from public databases as a strong and early MAMP-induced gene that is highly induced in roots (Hruz et al., 2008, Wyrsch et al., 2015); *WRKY11* (*WRKY DNA-BINDING PROTEIN 11*) is a representative of the WRKY transcription factor family, shown to mediate MAMP signaling and to be early-response genes themselves (Asai et al., 2002, Navarro et al., 2004). *MYB51* (*MYB DOMAIN PROTEIN 51*) was shown to be transcriptionally regulated by MAMPs and to control production of major *Arabidopsis* defense metabolites (Clay et al., 2009, Gigolashvili et al., 2007). We also generated *FRK1* (*FLG22-INDUCED RECEPTOR-LIKE KINASE 1*), a receptor-like protein of unknown function shown to be a strong and early MAMP-induced transcript (Asai et al., 2002, Boudsocq et al., 2010).

## Results

### flg22-Induced MAMP Responses Are Spatially Restricted in *Arabidopsis* Roots

**Figure 1:**
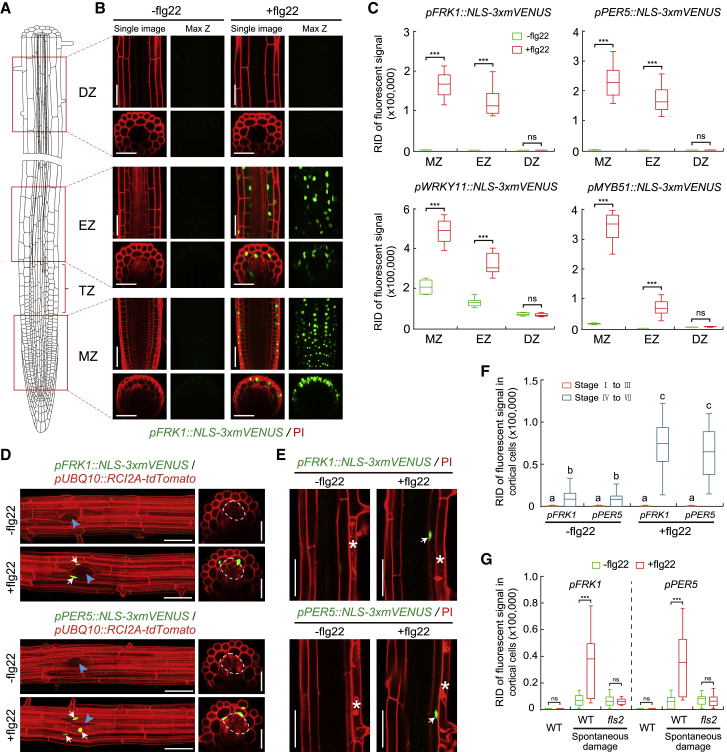
Flg22-Induced MAMP Responses Are Spatially Confined in *Arabidopsis* Roots (A) Schematic of a 6-day-old *Arabidopsis* root showing the different developmental zones. Three different zones were imaged: meristematic zone (MZ), elongation zone (EZ), and differentiation zone (DZ). TZ indicates the transition zone. (B) The expression pattern of one representative MAMP promoter marker lines (*pFRK1*) in response to 1 μM flg22 treatment for 6 h. Images correspond to the zones indicated in (A). Images in the differentiated zone were always taken at a distance of 25 endodermal cells after onset of cell elongation. In each treatment, single confocal section (single image, left) and maximal projections of z stacks (max z, right) are presented; median longitudinal and transverse (xz) section views are shown on the top and bottom, respectively. Nuclear-localized mVENUS signals (green) are co-visualized with propidium iodide (PI, red). Scale bar, 50 μm. (C) Quantitative analysis of mVENUS signal intensities of the four MAMP markers in the absence (−) or presence (+) of flg22. RID, raw intensity density. RID of total fluorescent signals in a single image is the sum of the RID of each nuclear signal in the imaged aera. RID of fluorescent signal of per nucleus = the size of the mVENUS signal area of a nucleus (number of pixels) × the average fluorescent intensity of the pixels for the nucleus. Boxplot centers show median (n = 12 roots). Asterisks (^∗∗∗^*p* < 0.001) indicate statistically significant differences between means by ANOVA and Tukey’s test analysis. ns, not significant. (D) MAMP responsiveness during lateral root primordium (LRP) formation. Images of stage IV lateral root in 8-day-old seedlings of double marker lines, highlighting plasma membrane of all root cells through *pUBQ10::RCI2A-tdTomato* expression (red) in addition to the MAMP responses (green). Maximum projections of longitudinal (left panel) and transverse sections (right panel) are shown. In transverse sections, a single red-channel image was overlaid with the green-channel maximum projection in order to obtain a clear plasma membrane outline. Arrows indicate cell nuclei with MAMP marker responses. The shape of emerged LRP is indicated by dotted circle in the orthogonal view, and site of emergence is indicated by a blue arrowhead in longitudinal maximum projections. Scale bar, 50 μm. (E) Spontaneous, non-induced cell death (asterisks) causes flg22 responsiveness (arrows) in neighboring cortical cell layer. Damaged epidermal cells are highlighted by PI staining. Scale bar, 50 μm. (F and G) Quantification of *FRK1* and *PER5* response to different developmental stages of lateral root emergence (F) and to non-induced (spontaneous) cell death in different backgrounds (G) with or without flg22 application. Boxplot centers show median (n = 10 roots). Different letters in (F) (*p* < 0.05) and asterisks in (G) (*p* < 0.001) indicate statistically significant differences between means by ANOVA and Tukey’s test analysis. ns, not significant. See also Figure S1.

**Figure S1:**
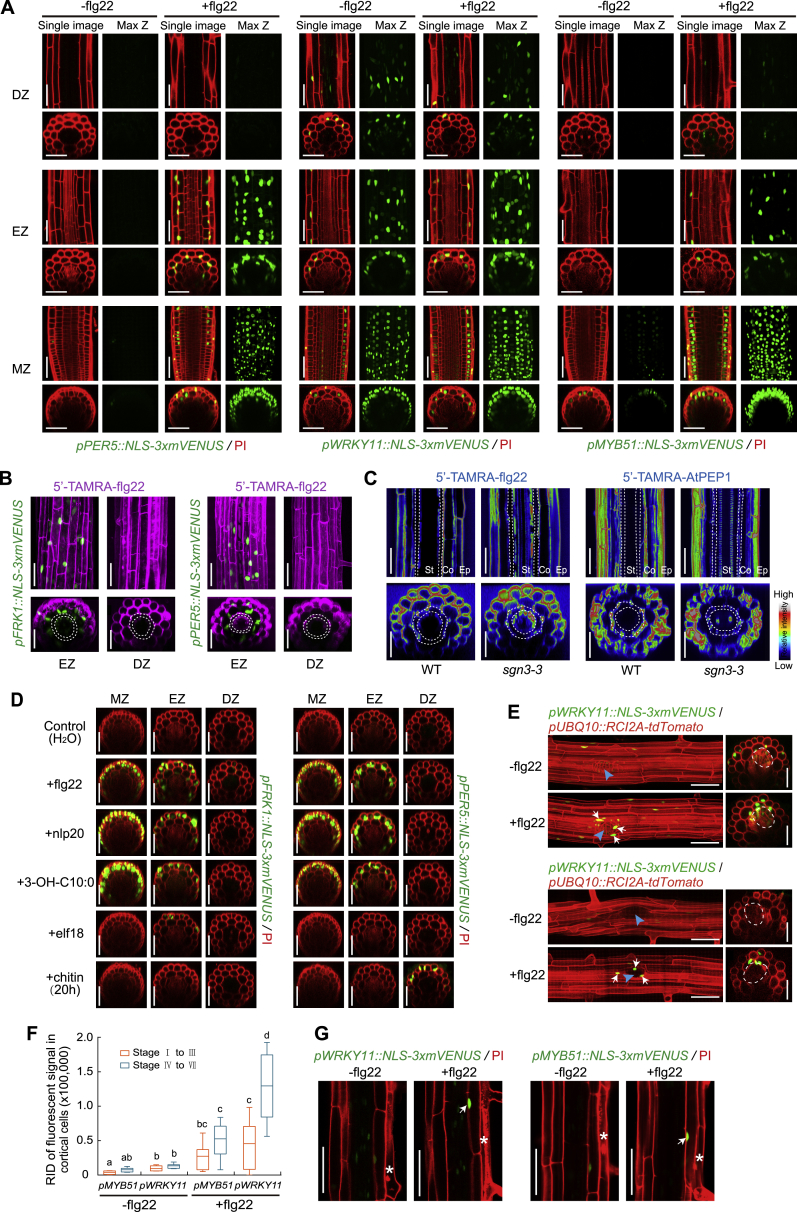
Localized MAMP Responsiveness in *Arabidopsis* Differentiated Roots, Related to Figure 1 (A) The expression pattern of three additional MAMP markers, *PER5*, *WRKY11* and *MYB51* in response to 1 μM flg22 treatment. Images taken are corresponding to the same position as in Figure 1A. Images in differentiated zone were always taken at a distance of 25 endodermal cells after onset of cell elongation. In each treatment, single confocal section (Single image, left panels) and maximal projections of Z stacks (Max Z, right panels) are presented; median longitudinal and transverse (xz) section views are shown in upper and bottom panels, respectively. Nuclear-localized mVENUS signals (green) are co-visualized with propidium iodide (PI, red). MZ, meristematic zone; EZ, elongation zone; DZ, differentiation zone. Scale bar, 50 μm. (B and C) Fluorescently-labeled peptide 5′-TAMRA-flg22 penetrates into roots through the apoplast. 5′-TAMRA-flg22 is functional and can activate distinct MAMP responses in the elongation zone (EZ) and differentiation zone (MZ) of the roots (B). Six-day-old roots were treated with 1 μM 5′-TAMRA-flg22 for 6h. Nuclear-localized mVENUS signals (green) co-visualized with TAMRA fluorescence (magenta). Representative images of the comparison of 5′-TAMRA-flg22 and 5′-TAMRA-AtPEP1 movement between WT and *sgn3-3* mutant background (C). Transverse and longitudinal view of the endodermal cell layer is indicated between dotted lines or circles. Note penetration of TAMRA fluorescence (royal LUT in ImageJ software) into the stele of *sgn3-3* mutant after 1 h peptide application. Maximum projections of longitudinal and transverse section views are shown in upper and bottom panel, respectively. Ep, epidermis; Co, cortex; St, stele. Scale bar, 50 μm. (D) Comparison of the response pattern of *FRK1* and *PER5* markers upon stimulation with different MAMPs. The chemicals were used at the following concentrations: 1 μM flg22, nlp20, 3-OH-C10:0, elf18 and 100 μg/ml chitin. All images were taken after 6 h treatment unless otherwise specified. Nuclear-localized mVENUS signals (green) are co-visualized with propidium iodide (PI, red). MZ, meristematic zone; EZ, elongation zone; DZ, differentiation zone. Scale bar, 50 μm. (E) flg22 responsiveness during lateral root primordium (LRP) formation. Images of stage IV of lateral root development of 8-day-old seedlings of double marker lines, highlighting plasma membrane of all root cells through *pUBQ10::RCI2A-tdTomato* expression (red) in addition to the MAMP responses (green, indicated by white arrows). The shape of emerged LRP is indicated by dotted circle in the orthogonal view, site of emergence is indicated by a blue arrowhead in longitudinal maximum projections. Image overlays done as described for Figure 1D. Scale bar, 50 μm. (F) Quantification of *MYB51* and *WRKY11* markers in response to different developmental stages of lateral root emergence with or without flg22 application. Boxplot centers show median (n = 10 roots). Different letters indicate statistically significant differences (*p* < 0.05, ANOVA and Tukey’s test). RID, see legend Figure 1C. (G) Spontaneous, non-induced cell death (asterisks) causes flg22 responsiveness (arrows) in neighboring cortical cell layer. Damaged differentiated epidermal cells are highlighted by PI staining.

Among the four MAMP markers generated, we found that *PER5* and *FRK1*, especially, displayed very low background before, and good induction upon, stimulation (Figures 1A–1C and S1A) (Poncini et al., 2017). For precise assignment of signals to specific cells and cell types, we generated double marker lines with a constitutively expressed, plasma membrane-targeted red fluorescent protein (Figure 1D). Alternatively, counterstaining with the red fluorescent cell wall stain propidium iodide (PI) was done.

Using these markers, we confirmed that MAMP-responses are confined to the root cap, transition/elongation zone, with an absent, or orders-of-magnitudes weaker, response in differentiated root parts, even at high doses of flg22 (1 μM) (Figures 1A–1C and S1A) (Millet et al., 2010). flg22, a peptide fragment of bacterial flagellin and a well-established elicitor in plants, was used as a prototypical MAMP (Felix et al., 1999). Lack of responses in differentiated roots is not due to a problem with peptide penetration, because the active, fluorescently labeled flg22 (TAMRA-flg22) fully penetrated the root until the endodermal diffusion barrier (Figures S1B and S1C). Thus, the absence of responses in the endodermis, cortex, and epidermis are not due to a block in MAMP penetration, while absence in the differentiated stele might be due to the endodermal diffusion barrier. The spatially restricted responses we observe are not observed only for flg22, because other MAMPs, such as nlp20 or a medium-chain 3-hydroxy fatty acid (3-OH-C10:0) (Böhm et al., 2014, Kutschera et al., 2019), display very similar response patterns (Figure S1D). elf18, another well-characterized bacterial MAMP (Kunze et al., 2004), showed very little response in roots overall, while the fungal chitin was the only MAMP that elicited some direct response in the differentiated zone.

Our high-resolution mapping of MAMP/flg22 responses revealed intriguing, spatially confined exceptions to the attenuated MAMP responses in differentiated roots. The first exception are emerging lateral roots, where adjacent cortical cells—that have become pushed, separated, possibly damaged, by the emerging primordium—consistently showed a strong response to MAMP treatment (Figures 1D, 1F, S1E, and S1F). The second exception we observed was a flg22 responsiveness in cells whose immediate neighbor had undergone sporadic cell death (Figures 1E, 1G, and S1G). Thus, differentiated roots have the capacity to respond to MAMPs and this responsiveness can be induced in a highly localized manner.

### Laser-Induced Cell Ablation Causes Localized MAMP Responsiveness in Roots

**Figure 2:**
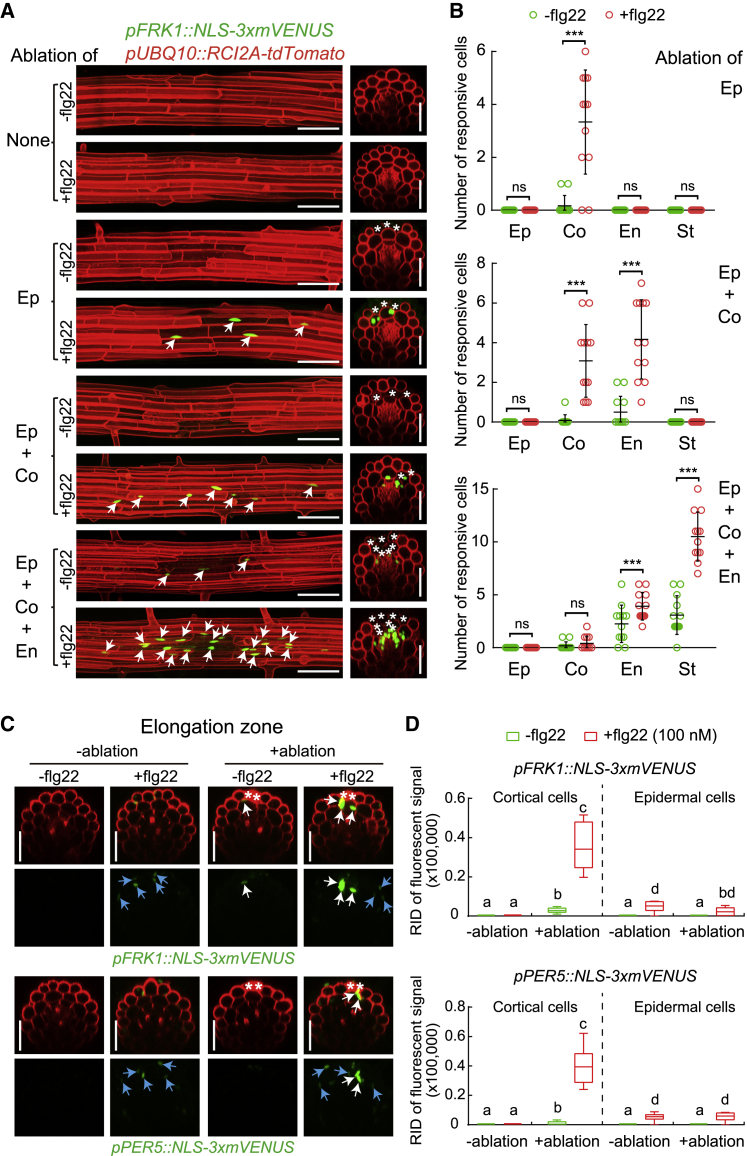
Restricted Cell Damage Causes Localized MAMP Responsiveness in Roots (A) In differentiated roots, laser ablation of different cell types induces localized *FRK1* response only in the presence of flg22 (+flg22, 1 μM, 6 h), but not on its own (−flg22). Nuclear-localized signals of *FRK1* reporter (green), co-visualized with the plasma membrane marker (see Figure 1D) (red). Images were taken at 25 endodermal cells after onset of cell elongation. Maximal projections of longitudinal and transverse sections are shown in left and right panels, respectively. White asterisks indicate laser-ablated cells. Arrows indicate *FRK1* responsive nuclei. RID, see legend Figure 1C. Scale bar, 50 μm. (B) Quantification of experiments shown in (A). Column scatterplot of the number of *FRK1* responsive cells in different cell types after laser ablation in the absence (green) or presence (red) of flg22. Each circle represents an individual laser ablation of one root (n = 12 roots). Graph depicts mean values and SD (error bars). Asterisks (*p* < 0.001) indicate statistically significant differences between means by ANOVA and Tukey’s test analysis. ns, not significant. Ep, epidermis; Co, cortex; En, endodermis; St, stele. (C) Damage of epidermal cells induces strong and localized *FRK1* and *PER5* response only in the presence of “suboptimal” (low) levels of flg22 (+flg22, 100 nM, 6 h), but not on its own (−flg22). Nuclear-localized signals of *FRK1* and *PER5* reporter (green) visualized alone (bottom panels, −PI) or co-visualized with PI staining (upper panels, +PI). White asterisks indicate laser-ablated cells. Arrows in white and blue indicate MAMP responsive nuclei by laser ablation and direct low level flg22 (100 nM) treatment in cortical and epidermal cells, respectively. Laser ablation and confocal images were taken at two or three cells just after onset of cell elongation. Scale bar, 50 μm. (D) RID quantification of experiments shown in (C). Boxplot centers show median (n = 12 roots). RID, raw intensity density. Different letters indicate statistically significant differences (*p* < 0.001) between means by ANOVA and Tukey’s test analysis. See also Figures S2, S3, and S5.

**Figure S2:**
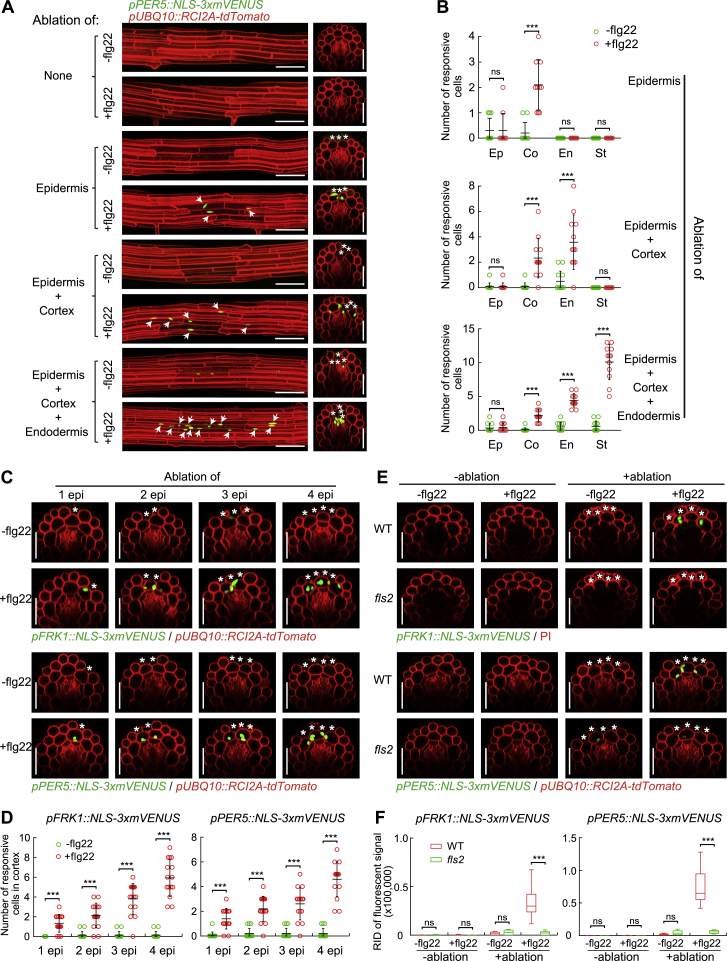
Laser Ablation-induced MAMP Responsiveness Rely on Cell Damage Extent and Functional FLS2, Related to Figure 2 (A and B) Representative images (A) and quantitative analysis by column scatterplot (B) of *PER5* responsiveness after laser ablation of different cell types in differentiated roots. Laser ablation and all images were at 25 endodermal cells after the onset of cell elongation. Nuclear-localized mVENUS signals for each *PRR* reporter (green) co-visualized with plasma membrane marker, *pUBQ10::RCIA2A-tdTomato* (red). Maximum projections of Z stack of mVENUS signals were combined with single red-channel images (see Figure 2A). White asterisks indicate laser-ablated cells. Arrows indicate *PER5* responsive nuclei. Scale bar, 50 μm. Each circle in (B) represents individual laser ablation event of one root (n = 12 roots). Graph depicts mean values and SD (error bars). Asterisks indicate significant differences between means by ANOVA and Tuckey’s test (*p* < 0.001). ns, not significant. Ep, epidermis; Co, cortex; En, endodermis; St, stele. Scale bar, 50 μm. (C and D) Representative images (C) and quantification by column scatterplot (D) of MAMP responsiveness after laser ablation of different epidermal cell numbers with or without flg22 for 6 h in differentiated roots. Nuclear-localized mVENUS signals of *FRK1* and *PER5* reporters (green) co-visualized with the plasma membrane marker, *pUBQ10::RCI2A:tdTomato* (red). White asterisk indicates damaged cell by laser ablation. Scale bar, 50 μm. Each circle in (D) represents individual laser ablation event (n = 12). Data represent mean values and SD (error bars). 1 epi, one epidermal cell; 2 epi, two epidermal cells; etc. (E and F) Orthogonal views (E) and RID quantification (F) of *FRK1* and *PER5* responsiveness in WT and *fls2* mutant background after combining without (-ablation) or with (+ablation) damage of epidermal cells in the absence or presence of flg22 for 6 h. Scale bar, 50 μm. Boxplot centers in (F) show median. Asterisks indicate significant differences between means (*p* < 0.001) by ANOVA and Tukey’s test analysis (n = 10 independent ablation events). ns, not significant. RID, see legend Figure 1C.

**Figure S3:**
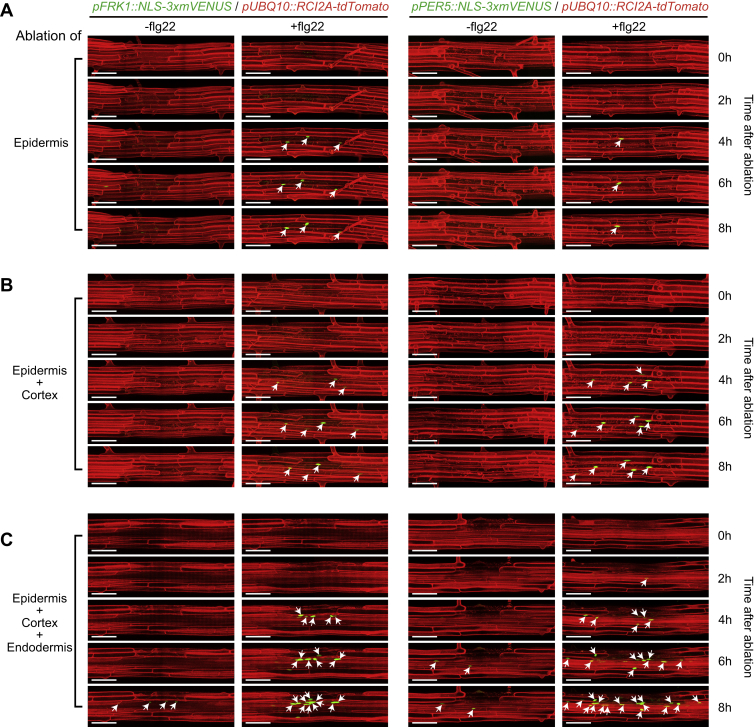
Time-Lapse Images of Ablation-Triggered Flg22 Responses, Related to Figure 2 (A-C) Real-time monitored MAMP responsiveness after laser ablation of different cell types in differentiated root cells. The combination of ablated cell types shown as following: (A) epidermal cells; (B) epidermal and cortical cells; (C) epidermal, cortical and endodermal cells. Nuclear-localized mVENUS signals of *FRK1* and *PER5* reporters (green) co-visualized with the plasma membrane marker, *pUBQ10::RCI2A:tdTomato* (red). Laser ablation and all images were at 25 endodermal cells after the onset of cell elongation. Maximal projections of Z stack of mVENUS signals and plasma membrane outline was merged together for longitudinal section view. White asterisk indicates damaged cell by laser ablation. Arrows indicate MAMP responsive nuclei. Scale bar, 50 μm.

The intriguing spatial association of MAMP responsiveness and neighbor-cell-death prompted us to induce reproducible and precise cellular damage and observe its effect on flg22 responsiveness. By ablating small clusters of distinct root cell types with a pulsed infrared laser, we observed a strong enhancement of flg22 responsiveness in immediately neighboring cell layers only (Figures 2A, 2B, S2A, and S2B). Importantly, ablation on its own led to no, or very little, induction of MAMP marker genes (Figures 2A, 2B, S2A, and S2B), showing that cellular damage per se is insufficient to induce a robust MAMP response. Already single-cell ablations induced flg22 responsiveness, but the effects became gradually more pronounced when more cells were ablated (Figures S2C and S2D), prompting us to use ablation of three or four cells as our standard. Time-lapse analysis showed that the earliest observable responses occurred at 4 h after flg22 treatment (Figure S3), leading us to use 6 h for most treatments. Introgression of our marker lines into an *fls2* mutant demonstrated a full dependency of the responses on a functional FLS2 receptor (Figures S2E and S2F). Interestingly, we observed directionality to damage induction, with inward-lying tissue layers generally responding the strongest. Cells of the stele responded strongly to flg22 upon epidermis, cortex, and endodermis ablation, while ablation of an epidermal cell did not cause flg22-responsiveness in epidermal neighbors (Figures 2A, 2B, S2A, and S2B). To explain the lack of responses in epidermal neighbors, one could postulate that mechanical stimulation is required for induction. Sudden pressure differences would only occur in cortex, but not in epidermal cells upon ablation, because epidermal cells do not experience counter-pressure from overlying cells. Another possibility might be that a collapse of plasmodesmatal integrity is perceived, and there are differences in quality and degree of plasmodesmatal connections between cortical and epidermal neighbors.

In the differentiated zone, absence of MAMP responsiveness without damage—even at high levels of flg22 (1 μM)—makes observation of the enhancement of MAMP responsiveness upon damage very obvious, leading to an essentially switch-like, qualitative change. Many commensal and root-pathogenic bacteria, however, preferentially colonize the root transition/elongation zone, which displays a direct response to high-doses of flg22, not requiring damage. Yet, when we used 100 nM of flg22, we saw only weak induction of MAMP responses in this zone (Figures 2C and 2D). In this situation of suboptimal stimulation, epidermal cell damage strongly enhanced response to flg22 in cortical cells, similar to the differentiation zone. Thus, although most easily observed in differentiated roots, damage-induced enhancement of MAMP responsiveness might be a wide-spread, possibly general, phenomenon in roots.

### Presence of DAMPs Alone Are Not Sufficient to Induce MAMP Responses

**Figure 3:**
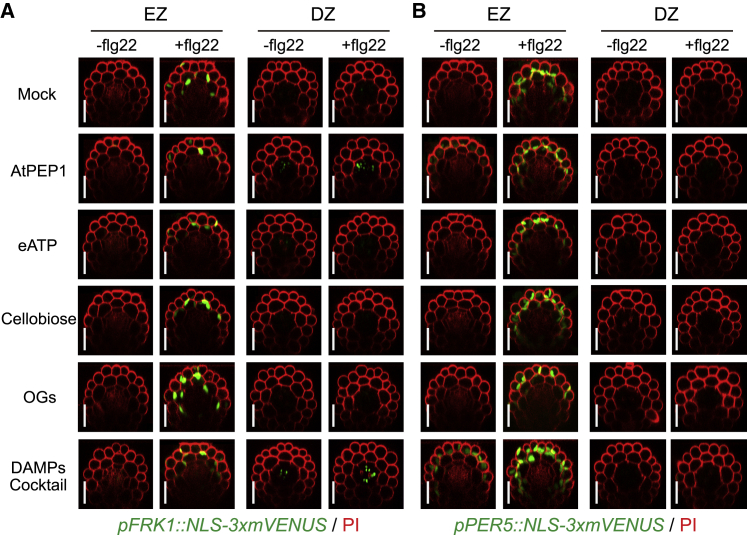
Presence of DAMPs Alone Are Not Sufficient to Induce MAMP Responses (A and B) Representative pictures of the expression pattern of *FRK1* (A) and *PER5* (B) markers in elongation zone (EZ) and differentiation zone (DZ) treated with a combination of flg22 and four types of DAMPs. Six-day-old roots were treated with each DAMP alone or combined with flg22 for 6 h. DAMPs cocktail is a mixture of all four tested DAMPs. The chemicals were used for treatment at the following concentrations: 1 μM flg22; 1 μM AtPEP1; 100 μM eATP; 100 μM cellobiose; 50 μg/mL OGs. Nuclear-localized mVENUS signals (green) co-visualized with PI counterstaining (red). Maximal projections of mVENUS signals and image overlaid in transverse sections done as described previously. Note that AtPEP1 leads a relatively weak *FRK1* response only in some differentiated stelar cells, which is not the case for *PER5* marker, rather than in cortical or endodermal cells that we observed upon actual cellular damage and that DAMPs cocktail, but not single DAMP, is able to activate a weak *PER5* responsiveness in the elongation zone. Scale bar, 50 μm.

How cellular damage is perceived by neighboring cells is not well understood, but one important element is thought to be the release of damage-associated molecular patterns (DAMPs), which can be abundant, but largely cytosolic molecules such as adenosine triphosphate (ATP), or small peptides, such as AtPEP1 (Roux and Steinebrunner, 2007, Toyota et al., 2018, Hander et al., 2019). In plants, cell wall-breakdown products, such as oligogalacturonides (OGs) and cellobiose are additionally acting as DAMPs (Boller and Felix, 2009, Lotze et al., 2007, Souza et al., 2017). Interestingly, even when applied systemically at high concentrations, either individually or as a cocktail, DAMPs alone were not able to induce the strong and consistent flg22 responsiveness that we observe upon actual cellular damage (Figures 3A and 3B). AtPEP1 treatment alone caused some slight induction of *FRK1*—but not *PER5* responsiveness—in the stele, but could not induce any MAMP responsiveness in differentiated outer cell layers. This suggests that perception of neighbor cell damage is more complex than a simple presence of DAMPs, relying on additional cues, possibly ion and osmolyte release or mechanical stress, caused by cellular disintegration.

### MAMP Receptor Expression Is Induced by Cell Ablation and Is Sufficient to Induce Responsiveness

**Figure 4:**
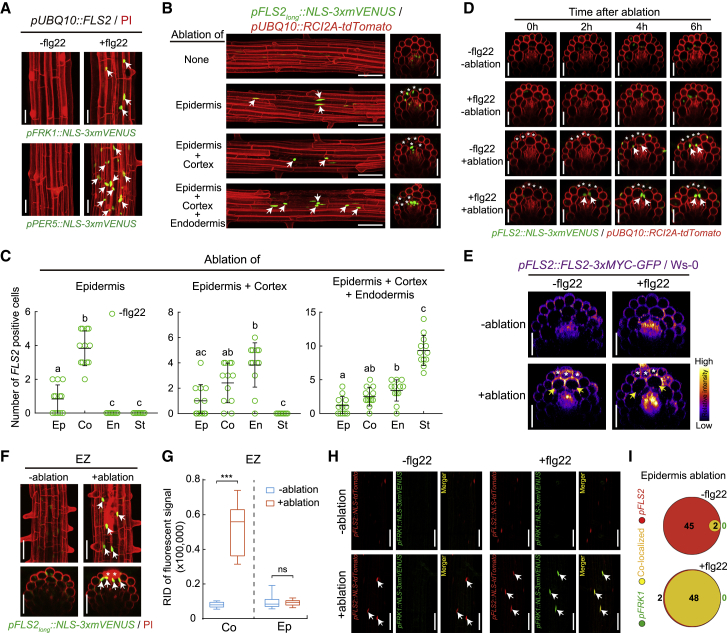
Localized *FLS2* Expression Induced by Neighbor Cell Death (A) Expression of *FRK1* and *PER5* marker (green) with or without flg22 treatment (1 μM, 6 h) in differentiated zone (DZ) of a *pUBQ10::FLS2* transgenic background. Marker line was counterstained with PI (red). Arrows indicate MAMP responsive nuclei. Scale bar, 50 μm. (B) Laser ablation of different cell types (without flg22 treatment) induces localized *FLS2* expression in 6-day-old differentiated roots. Nuclear-localized mVENUS signals of *FLS2* promoter marker (green) co-visualized with plasma membrane marker (red). Images overlaid was done as described before and pictures were taken at 25 endodermal cells after onset of cell elongation. Asterisks highlight laser-ablated cells and arrows indicate *FLS2*-positive nuclei. Scale bar, 50 μm. (C) Quantification of the number of *FLS2*-positive cells in different cell types shown in (B). Column scatterplot of the number of *FRK1* responsive cells after laser ablation in the absence (green) or presence (red) of flg22. Each circle represents an individual laser ablation of one root (n = 12 roots). Graph depicts mean values and SD (error bars). Asterisks (*p* < 0.001) indicate statistically significant differences between means by ANOVA and Tukey’s test analysis. ns, not significant. Ep, epidermis; Co, cortex; En, endodermis; St, stele. (D) Real-time monitored *FLS2* induction after laser ablation of differentiated epidermal cells with or without flg22 application in orthogonal view. Asterisks and arrows highlight laser-ablated cells and *FLS2*-positive nuclei, respectively. Scale bar, 50 μm. (E) Maximal projections of orthogonal view of accumulation of FLS2-fused protein (FLS2-GFP) by ablation of epidermal cells. Yellow arrows highlight upregulated FLS2-GFP fluorescence (fire LUT of ImageJ software) in neighboring cortical cells. White asterisks indicate damaged cell by laser ablation. Scale bar, 50 μm. (F and G) Cell damage activates localized *FLS2* expression level in the undifferentiated zone. In (F), nuclear-localized signals of *FLS2* (green) co-visualized with the PI staining (red), and white arrows highlighted positive nuclei neighboring damaged epidermal cells. Boxplot centers in (G) show median (n = 12 roots). RID, raw intensity density, see legend Figure 1C. Asterisks letters indicate statistically significant differences (^∗∗∗^*p <* 0.001) between means by ANOVA and Tukey’s test analysis. ns, not significant. EZ, elongation zone; Ep, epidermis; Co, cortex. Scale bar, 50 μm. (H) *FLS2* expression was co-visualized with *FRK1* expression in cortical cells after laser ablation of adjacent epidermal cells. *FLS2* promoter-driven nuclear tdTomato signal (red) and nuclear MAMP reporter signal (green) are co-localizing (yellow) in the presence of flg22 application for 6 h. Arrows indicate MAMP responsive or/and *FLS2*-positive nuclei. Scale bar, 50 μm. (I) Venn diagrams showing the number of co-localized cells in cortex (yellow) of *FLS2*-positive (red) and MAMP-responsive cells (green) caused by laser-ablation of epidermal cells. The total cell number for each marker was added from 10 independent ablation events. The size of each circle reflects relative cell numbers. See also Figures S4 and S5.

**Figure S4:**
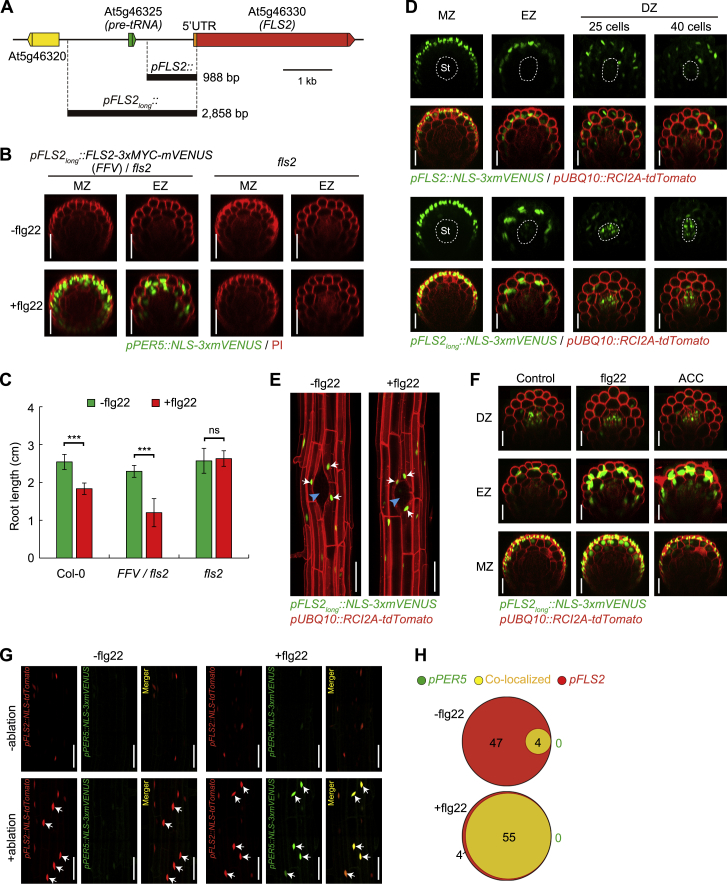
*FLS2* Expression Patterns in *Arabidopsis* Roots, Related to Figure 4 (A) Schematic map of two *FLS2* promoters with different length and neighboring genome region. The shorter promoter, *pFLS2* was cloned from original study (Gómez-Gómez and Boller, 2000). The longer one, *pFLS2*_*long*_ promoter, covers the sequence of *pFLS2*, then extending to the upstream region of another neighboring gene *At5g46325*, a putative *pre-tRNA* gene. Color box: gene locus; black line: intergenic sequence. (B and C) The longer promoter *pFLS2*_*long*_, driving an FLS2-mVENUS construct, was shown to rescue MAMP responses in *fls2* mutant background. Complementation analysis of *PER5* maker induction (B) and root growth inhibition (C) in response to flg22 treatment. Asterisks in (C) indicate statistically significant differences (*p* < 0.001) between means by ANOVA and Tukey’s test analysis. ns, not significant. MZ, meristematic zone; EZ, elongation zone. Scale bar, 25 μm. (D) Comparison of the expression patterns between the two promoters in different zones of the root. Nuclear-localized *FLS2* mVENUS signals only (green, upper panel) or co-visualized with plasma membrane marker (red, bottom panel). For differentiation zone (DZ), longitudinal sections of images were taken at 25 or 40 endodermal cell numbers after the onset of cell elongation, respectively. Dotted circles indicate the stele (St). Scale bar, 25 μm. (E) Localized *FLS2* induction during lateral root primordium (LRP) formation without (-) or with (+) flg22. Maximal projections of longitudinal sections were showing the stage IV of lateral root development of eight-day-old seedlings. Site of emergence is indicated by a blue arrowhead. Arrows indicate *FLS2*-induced nuclei. Scale bar, 50 μm. (F) Activity of *pFLS2_long_* promoter under flg22 (1 µM) or ACC (10 µM) induction condition for 6 h in different zones of the root. Scale bar, 25 µm. (G) *FLS2* expression were co-visualized with *PER5* expression in cortical cells after laser ablation of adjacent epidermal cells. *FLS2* promoter-driven nuclear tdTomato signal (red) and nuclear MAMP reporter signals (green) are co-localizing (yellow) in the presence of flg22 application for 6 h. Arrows indicate MAMP responsive or/and *FLS2*-positive nuclei. Scale bar, 50 μm. (H) Venn diagrams showing the number of co-localized cells in cortex (yellow) of *FLS2*-positive (red) and *PER5*-responsive cells (green) caused by laser-ablation of epidermal cells. The total cell number for each marker was accumulated from 10 independent ablation events. The relative size of each circle reflects counted cell numbers.

We found that expressing the MAMP receptor FLS2 under a constitutive *UBIQUITIN 10* promoter (*pUBQ10*) was sufficient to install responsiveness to flg22 in differentiated outer root cell layers (Figure 4A). This indicates that FLS2 itself is the only component restricting the ability of differentiated root cells to respond to flg22, implying that all other necessary downstream components (such as BRI1-associated kinase [BAK1], Botrytis-induced kinase [BIK1], MAPKs, WRKYs, etc.) are present. This fits with earlier observations of MAMP receptor mis-expression in other organs or species (Lacombe et al., 2010, Wyrsch et al., 2015). Consequently, we wanted to also monitor *FLS2* expression at single-cell resolution after damage. The currently used *FLS2* promoter complements *fls2* (Zipfel et al., 2004) and roughly matches the spatial patterns of MAMP responses (our work and [Beck et al., [2014]). However, the promoter is of small size (less than 1,000 bp), shows important line-to-line variability and in some cases does not match with MAMP responses (Beck et al., 2014). We therefore additionally generated a longer promoter line (*pFLS2*_*long*_) (Figure S4A), which showed less variability and an average pattern that is largely consistent with the described flg22-induced MAMP responses (Figure S4D), i.e., responses adjacent to emerging lateral roots or enhancement of responses to ethylene (Figures S4E and S4F). *FLS2* expression from this longer promoter fragment also complemented the absence of flg22 responses in *fls2* background (Figures S4B and S4C).

In contrast to the MAMP response markers, we found that *FLS2* is transcriptionally activated upon wounding alone, both in differentiation and elongation zone of the root (Figures 4B–4D, 4F, and 4G), readily explaining how cells can become responsive upon wounding. Indeed, the timing and spatial extent of *FLS2* upregulation matched the observed pattern of MAMP responsiveness (compare Figures 4B–4D and 4F with Figures 2A–2D and S3, respectively). We confirmed that, although less easily quantifiable, a local upregulation of FLS2 protein could also be observed using *pFLS2::FLS2-GFP* reporter line (Figure 4E). To fully correlate local *FLS2* activation upon damage with MAMP responsiveness, we generated double marker lines of *pFLS2::NLS-tdTomato* and mVENUS MAMP reporters and found that the near-totality of neighboring MAMP responsive cells were also positive for *FLS2* expression when treated with flg22 upon ablation (Figures 4H, 4I, S4G, and S4H). Previously, *pFLS2::GUS* reporter lines showed signal in regions around large-scale wound sites, but relevance for MAMP signaling was not established at the time (Beck et al., 2014). Our co-visualization of receptor expression and MAMP responses now additionally reveals that transcriptional MAMP responses can be strictly cell autonomous, allowing for a very fine-grained activation of immunity. This degree of spatial specificity is surprising, considering that flg22 stimulation was shown to induce ROS production, depolarization, and even propagating calcium waves, all of which have the potential to induce non-cell autonomous responses (Jeworutzki et al., 2010, Keinath et al., 2015).

### Induction of MAMP Responsiveness by Damage Does Not Require Ethylene Signaling

*FLS2* expression is also known to strongly depend on ethylene (Boutrot et al., 2010, Mersmann et al., 2010) and recent work from our group demonstrated that single cell ablation causes regional induction of ethylene production (Marhavý et al., 2019). Although the spatial patterns of ethylene production reporters upon ablation (extending over many cellular distances, mainly in the stele, no induction of immediate neighbors) did not match the observed *FLS2* induction pattern (Marhavý et al., 2019), we nonetheless tested whether *FLS2* upregulation after damage depended on ethylene.

**Figure S5:**
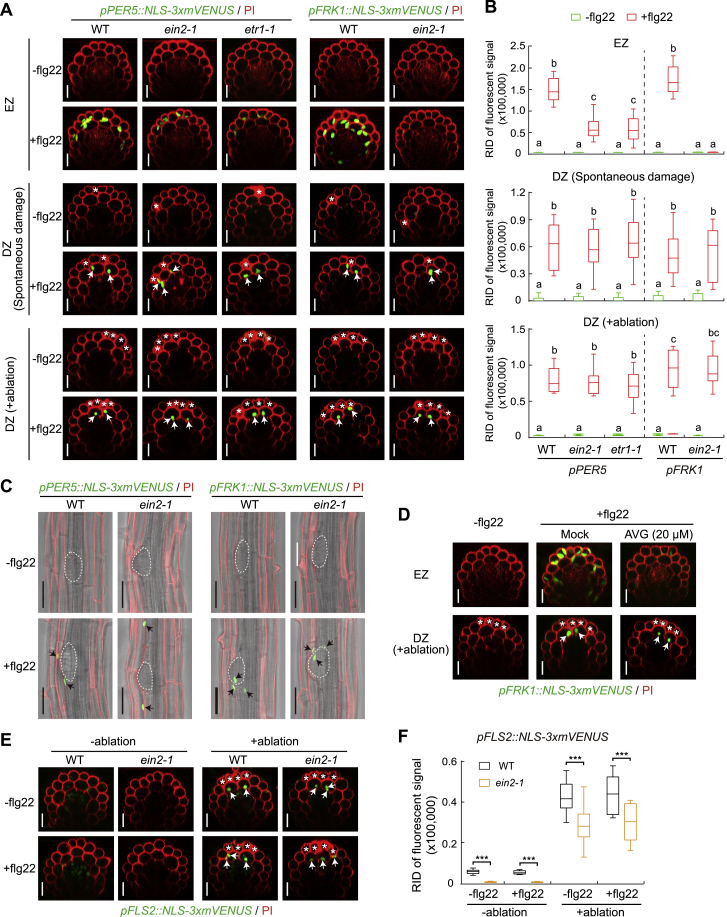
Unlocking of Flg22 Responsiveness by Cell Damage Is Independent of Ethylene Signaling, Related to Figures 2 and 4 (A and B) Representative images (A) and quantitative analysis by boxplot chart (B) of *PER5* and *FRK1* responsiveness without (-) or with (+) flg22 treatment in WT and ethylene insensitive mutants, *ein2-1* and *etr1-1*, elongating roots (upper panel), spontaneously damaged roots (middle panel) and laser-ablated differentiated roots (bottom panel). Note MAMP responsiveness in elongation zone is partially or completely dependent on ethylene signaling as MAMP fluorescent signals, compared to WT, are highly decreased (*PER5*) or fully abolished (*FRK1*) in ethylene insensitive mutants after flg22 application for 6 h. Nuclear-localized mVENUS signals (green) co-visualized with PI counterstaining (red). White asterisks indicate damaged cells. In (B), boxplot centers show median (n = 12 roots). Different letters (*p* < 0.001) indicate statistically significant differences between means by ANOVA and Tukey’s test analysis. RID, raw intensity density. Scale bar, 50 μm. (C) Longitudinal view of maximum projection of MAMP responsiveness in the absence (-) or presence (+) of flg22 in WT and *ein2-1* mutant LRP formation site. Emerged LRP shape is highlighted by dotted circle in the bright-field background (gray). Black arrows indicate responsive nuclei. Scale bar, 50 μm. (D) Maximum projection of *FRK1* reporter in elongated cells (upper panel) or laser-ablated differentiated cells (bottom panel) pre-treated with ethylene biosynthesis inhibitor, 2-aminoethoxyvinyl glycine (AVG) for 2 h. Scale bar, 50 μm. (E and F) Confocal images (E) and RID quantitative analysis (F) of *FLS2* induction without (-ablation) or with (+ablation) laser-damaged epidermal cells in comparison between WT and *ein2-1* differentiated roots. Laser ablations were performed at 25 endodermal cells after onset of cell elongation. White asterisks indicate damaged cells. Boxplot centers in (F) show median (n = 12 roots). Asterisks (*p* < 0.001) indicate statistically significant differences between means by ANOVA and Tukey’s test analysis. RID, see legend Figure 1C. Scale bar, 50 μm.

By combining *FLS2* reporter and MAMP markers in strong ethylene-insensitive mutants, *ein2-1* and *etr1-1*, we could observe a very strong dependency of MAMP responses on ethylene signaling in the elongation zone (Figures S5A and S5B), consistent with a previous study (Millet et al., 2010). However, both sporadic and laser-induced cell damage were still able to induce MAMP responsiveness, independently of ethylene signaling (Figures S5A and S5B). This also applies to lateral root emergence, where cortical cells showed upregulation of *FLS2* expression independently of ethylene signaling (Figure S5C). Treating wild-type MAMP marker lines with ethylene biosynthesis inhibitor corroborated these results (Figure S5D). Consequently, induction of *FLS2* expression itself upon damage was also found to be fully independent on ethylene signaling, although the basal expression levels in the untreated controls were strongly reduced (Figures S5E and S5F). These findings now provide a rationale for earlier observations noting that impaired flg22 signaling in ethylene mutants is not observed in assays involving dissected (wounded) tissues (Mersmann et al., 2010). Importantly, we establish an abiotic stress input into immune signaling that appears to work fully independently of the important stress hormone ethylene.

### Casparian Strips Compartmentalize flg22 Responses in Differentiated Roots

**Figure 5:**
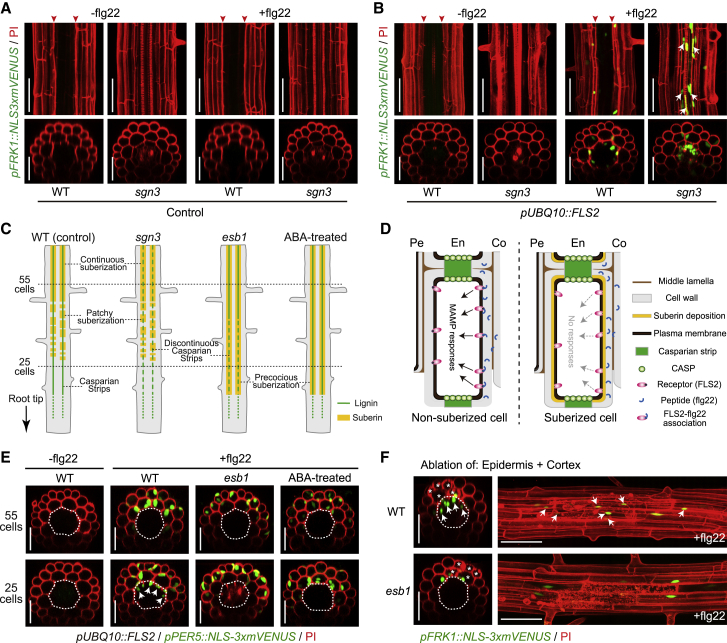
Endodermal Barriers Compartmentalize MAMP Responses in Differentiated Roots (A and B) Expression pattern of *FRK1* marker in the absence or presence of flg22 in the differentiated zone of WT and endodermal barrier-defective *sgn3-3* roots in Col-0 (A) and *pUBQ10::FLS2* lines (B). Arrowheads indicate site of PI penetration block by the Casparian strips. Note the penetration of PI signals (red) into the stele in *sgn3-3* mutants, revealing their barrier defects. Arrows in (B) indicate MAMP-responsive (*FRK1*-positive) nuclei (green) in the stele of *sgn3-3*. Maximal projections of confocal image stacks were taken at 25 endodermal cells after the onset of cell elongation. Nuclear-localized mVENUS signals (green) counterstained with PI. Scale bar, 50 μm. (C) Schematic view of the two endodermal barriers—Casparian strips and suberin lamella—in different backgrounds (WT, *sgn3-3*, and *esb1-1* mutants) and ABA treatment. Lignin and suberin deposition in the endodermis are represented by green and yellow lines, respectively. (D) Schematic depicting the putative role of suberin lamellae in restricting receptor-peptide recognition on the cell surface. Primary stage and secondary stage of endodermal differentiation are presented by non-suberized (left) and suberized (right) endodermal cells, respectively. In non-suberized cells, peptides can access to the endodermal plasma membrane through apoplastic movement. The resulting plasma membrane-localized receptor-peptide (FLS2-flg22) association is capable of activating downstream MAMP responses inside the cell. By contrast, in suberized cells, direct MAMP signal perception on the cell surface is blocked by the presence of suberin lamellae between plasma membranes and primary cell walls of endodermal cells, interrupting the downstream responses. (E) Representative images depicting expression of *PER5* reporter combined with *FLS2* constitutive expression line (*pUBQ10::FLS2*) in different backgrounds (WT and *esb1-1* mutant) or pre-treatment with ABA (1 μM, 18 h). Dotted circles and arrows indicate the boundary between endodermal and cortical layers, and endodermal *PER5* responsive nuclei, respectively. Scale bar, 50 μm. (F) Co-ablation of epidermal and cortical cells triggers responsiveness to flg22 in differentiated endodermal cells of WT, but not in the precociously suberizing *esb1-1* mutant. White asterisks indicate damaged cells by laser ablation. Maximal projections of confocal image stacks. Image overlays done as described for Figure 1D. Dotted circles and arrows indicate the boundary between endodermal and cortical layer, and endodermal *FRK1* responsive nuclei, respectively. Scale bar, 50 μm. See also Figure S6.

In light of the comparatively high expression of *FLS2* in the stele of differentiated roots, we tested whether a mutant defective in Casparian strips, the extracellular diffusion barrier in roots (Geldner, 2013), would display flg22 responsiveness, because of penetration of flg22 into the stele. Indeed, fluorescently labeled flg22 is blocked by the Casparian strip and penetrates into the stele in the barrier mutant (*schengen3-3* [*sgn3-3*]) (Figure S1F). Yet, to our surprise, no flg22 response was observed in the stele of a *sgn3* mutant with endogenous *FLS2* expression (Figure 5A). However, when a constitutively expressing *pUBQ10::FLS2* line was used, a strong flg22 response could be observed in the stele of the endodermal barrier mutant, but not of wild-type (Figures 5B and 5C). This result illustrates the ability of the Casparian strip to compartmentalize perception of immune peptides within the root. Interestingly, however, the wild-type, steady-state levels of *FLS2* expression that we observe in the stele are apparently insufficient to cause MAMP-responsiveness, while enhanced receptor expression from the *UBQ10* promoter is sufficient to install responsiveness. This suggests a thresholded relationship between *FLS2* expression and flg22-dependent transcriptional output.

### Suberin Lamellae Interfere with flg22 Perception in the Endodermis

**Figure S6:**
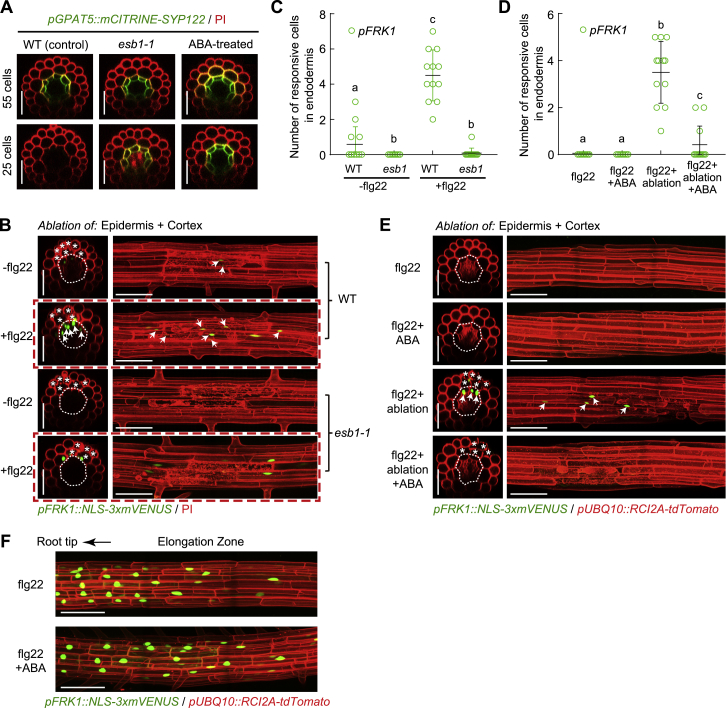
Suberin Lamellae Interfere with flg22 Perception in the Endodermis, Related to Figure 5 (A) Suberin plasma membrane marker *pGPAT5::mCITRINE-SYP122* expression (green) along the root developmental stages in different backgrounds (WT and *esb1-1* mutant) or treated with 1 μM ABA (WT background) prior to observation. The *GPAT5* reporter line counterstained with PI (red). Images were taken at 25 or 55 endodermal cell numbers after the onset of cell elongation, respectively. (B and C) Representative images (B) and quantitative analysis by column scatterplot (C) of co-ablation of epidermal and cortical cells triggers responsiveness to flg22 in differentiated endodermal cells of WT, but not in the precociously-suberizing *esb1-1* mutant (B). Nuclear-localized mVENUS signals (green) co-visualized with PI staining or plasma membrane marker (red). Maximum projections of transverse (left panel) and longitudinal sections (right panel) are shown. Arrows represent endodermal *FRK1*-responsive cell nuclei. White asterisks indicate damaged cells by laser ablation, taken at 25 endodermal cells after the onset of cell elongation. Note images in red dotted box were used for Figure 5D. Each circle in (C) represents individual laser ablation event of one root (n = 12 roots). Values are means ± SD. Individual letters indicate statistically significant differences (*p* < 0.001, ANOVA and Tukey’s test). (D and E) Quantification (D) and images (E) of co-ablation of epidermal and cortical cells triggers responsiveness to flg22 in differentiated endodermal cells of non-treated control, but not in ABA pre-treated roots (E). Each circle in (D) represents individual laser ablation event of one root (n = 12 roots). Values are means ± SD. Individual letters indicate statistically significant differences (*p* < 0.001, ANOVA and Tukey’s test). (F) ABA treatment did not affect MAMP responses in elongating root cells. Six-day-old roots were pre-treated with 1 μM ABA prior to flg22 application for 6h. Pictures are maximum projections of confocal Z stacks. ABA pre-treatment in (D-F) was performed for 18 h. Scale bar, 50 μm.

While the Casparian strip functions to block extracellular diffusion of substances (e.g., microbial patterns) into the stele, a second cell wall modification—endodermal suberin lamellae—eventually surrounds the entire endodermis and is thought to inhibit uptake of molecules into the endodermis, because the hydrophobic suberin layer does not allow molecules from the cell wall to reach the endodermal plasma membrane (Figures 5C and 5D) (Barberon et al., 2016). We therefore wanted to see whether suberization interferes with the ability of endodermal cells to perceive flg22. Indeed, we found that early differentiated endodermis (25 cells after onset of elongation, *non-suberized*) still respond to flg22 in a *pUBQ10::FLS2* line, while they are unresponsive in older endodermal cells (55 cells after onset of elongation, *suberized*) (Figures 5C and 5E). We confirmed absence and presence of suberin at 25 and 55 cells, respectively, using a previously established suberization marker, *pGPAT5::mCITRINE-SYP122* (Barberon et al., 2016, Naseer et al., 2012) (Figure S6A). By inducing precocious and enhanced suberization by two different mechanisms, using either the *enhanced suberin 1 (esb1*) mutant or treatment with abscisic acid (ABA) (Barberon et al., 2016, Hosmani et al., 2013, Wang et al., 2019), flg22 responsiveness was suppressed in early endodermis (25 cells) (Figures 5C and 5E), demonstrating that protective suberization of a cell is incompatible with continued perception of microbial patterns (Figure 5D). This suppression of endodermal responses by suberization could not only be observed in the constitutive *FLS2*-expressing line, but also with endogenously expressed *FLS2*, after ablation of epidermis and cortex. In this case again, we found that endodermal flg22 responses, observed in early differentiated cells, were abrogated in *esb1* (Figures 5F, S6B, and S6C) or upon ABA treatment (Figures S6D and S6E). We ascertained that ABA does not cause a general suppression of MAMP responses, because responses in the root elongation zone are maintained upon ABA treatment (Figure S6F).

### Cell Damage Activates Expression of Multiple Pattern-Recognition Receptors

**Figure 6:**
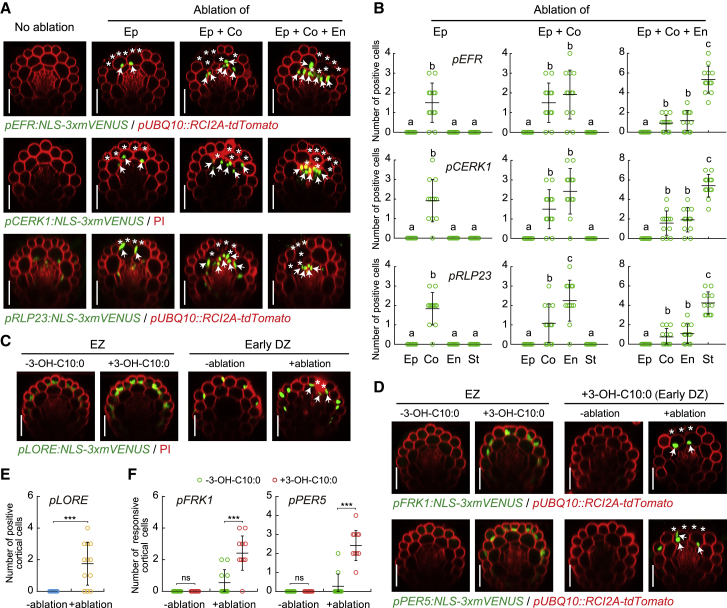
Cell Damage Activates Expression of Multiple Pattern-Recognition Receptors (A and B) Representative images (A) and quantitative analysis by column scatterplots (B) of promoter activation of three additional *PRRs* after laser ablation of different cell types in differentiated roots. Nuclear-localized mVENUS signals for each *PRR* reporter (green) co-visualized with plasma membrane marker, *pUBQ10::RCIA2A-tdTomato* or PI counterstaining (red). Maximum projections of Z stack of mVENUS signals were combined with single red-channel images. White asterisks indicate laser-ablated cells. Arrows indicate *PRR* promoter-positive nuclei. Each circle in (B) represents individual laser ablation event of one root (n = 12 roots). Graph depicts mean values and SD (error bars). Different letters indicate significant differences between means by ANOVA and Tuckey’s test (*p* < 0.001). Ep, epidermis; Co, cortex; En, endodermis; St, stele. Scale bar, 50 μm. (C) The expression pattern of another *PRR* reporter, *LORE* in response to 1 μM 3-OH-C10:0 treatment in the elongation zone (EZ) and cell ablation in the early differentiation zone (DZ), respectively. Maximum projections of z stack of mVENUS signals were combined with single red-channel images. Scale bar, 50 μm. (D) The expression pattern of MAMP reporters in response to 3-OH-C10:0 treatment in the elongation zone or combined with ablation in the early differentiation zone. White asterisks and arrows in (C) and (D) indicate laser-ablated cells and reporters positive/responsive nuclei in cortical cells, respectively. Scale bar, 50 μm. (E and F) Quantitative analysis by column scatterplot of *LORE* reporter (E) and MAMP responsiveness (F) in the absence (−) or presence (+) of laser ablation in 3-hydroxydecanoic acid treated (+3-OH-C10:0) or untreated (−3-OH-C10:0) roots. Each circle represents individual laser ablation event of one root (n = 12 roots). Graph depicts mean values and SD (error bars). Asterisks indicate significant differences between means by ANOVA and Tuckey’s test (*p* < 0.001).

We then broadened our observations based on FLS2 to other MAMP receptors by establishing transcriptional reporter lines for three additional *PRR*s, the *EF-TU RECEPTOR* (*EFR*) (Zipfel et al., 2006), the *CHITIN ELICITOR RECEPTOR KINASE 1* (*CERK1*) (Miya et al., 2007), as well as the nlp20 receptor *RECEPTOR-LIKE PROTEIN 23 (RLP23*) (Albert et al., 2015). In all three cases, a very similar, localized upregulation of receptor expression upon laser-induced cell damage was observed (Figures 6A and 6B), suggesting that cell damage leads to a rather generalized upregulation of response capacity to MAMPs.

We then used an independent MAMP, 3-OH-C10:0, the newly described ligand for the *LIPOOLIGOSACCHARIDE-SPECIFIC REDUCED ELICITATION* (*LORE*) receptor kinase (Ranf et al., 2015). Similar to the other *PRRs*, *LORE* expression is strongly induced upon damage in the early differentiated cells (Figures 6C and 6E). 3-OH-C10:0 elicits direct MAMP responses in the elongation zone, but not in the differentiation zone, similar to flg22 (Figure 6D). More importantly, upon damage, a strong enhancement of responses to 3-OH-C10:0 was observed in the early differentiation zone (Figures 6D and 6F), showing that the observed damage-gating of MAMP responses is not restricted to flg22-FLS2 module, but is also observed for a non-peptidic, conserved bacterial pattern, perceived by a non-LRR type receptor.

### Local Gating of Immune Responses by Damage in Root-Bacteria Interactions

**Figure 7:**
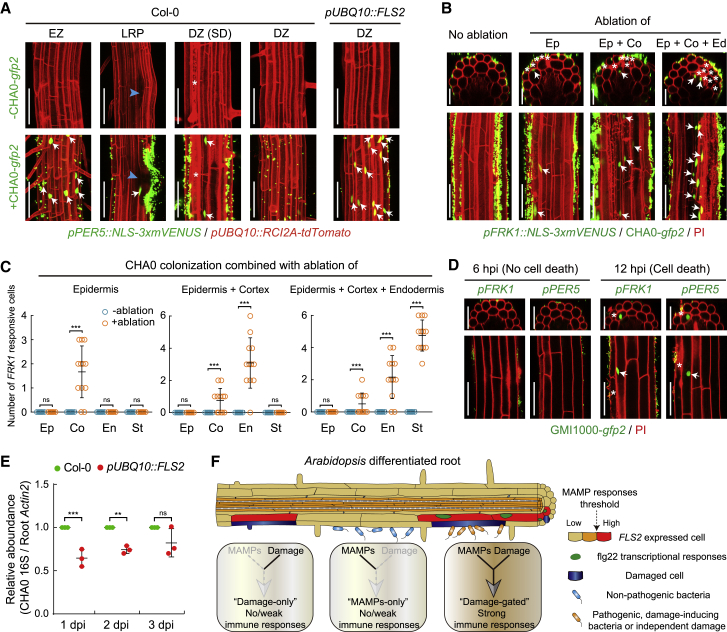
Local Gating of Immune Responses by Damage in Root-Bacteria Interaction (A) Comparison of *PER5* responsiveness in different developmental zones of control (Col-0) and *FLS2*-overexpressing line (*pUBQ10::FLS2*) in the absence (−CHA0-*gfp2*) or presence (+CHA0-*gfp2*) of bacterial colonization for 9h. MZ, meristematic zone; EZ, elongation zone; DZ, differentiation zone; LRP, lateral root primordium. A blue arrowhead indicates the site of lateral root emergence. White asterisks and arrows indicate non-induced damaged cells and *PER5* responsive nuclei, respectively. Scale bar, 50 μm. (B) Laser-induced cell damages can cause MAMP responsiveness (as *FRK1* marker-positive cells) in differentiated roots in response to non-pathogenic CHA0 microbe colonization. Laser ablation was performed on indicated cell layer(s) followed by 9 h colonization by CHA0-*gfp2* strain (OD_600_ = 0.1). Laser-ablated cells are indicated by white asterisks. Arrows indicate localized *FRK1* responses (green), easily distinguished by size and shape from green fluorescent bacteria. Counterstained with PI (red). Image overlays done as described before. Scale bar, 50 μm. (C) Quantification of experiments shown in (A). Column scatterplots of the number of *FRK1* responsive cells in different cell types without (blue, −ablation) or with (orange, +ablation) laser damage of different cell layer(s). Each circle represents an individual laser ablation event of one root (n = 12 roots). Graph depicts mean values and SD (error bars). Asterisks indicate significant differences between means (^∗∗∗^*p* < 0.001) by ANOVA and Tukey’s test analysis. ns, not significant. Ep, epidermis; Co, cortex; En, endodermis; St, stele. (D) Local MAMP responses could also be observed in cells adjacent to damaged cells, observed 12 h post infection (hpi) with the root pathogenic bacteria GMI1000-*gfp2*. By contrast, upon infection with GMI1000 for short time course (6 hpi), no cell death, and no MAMP response were observed in differentiated cortical cells. Damaged cells associated with GMI1000 infection are indicated by white asterisks. Arrows indicate localized MAMP responses (green), counterstained with PI (red). Scale bar, 50 μm. (E) Quantitative measurement of relative CHA0 abundance in Col-0 and *pUBQ10::FLS2* roots at indicated colonization time point. Roots colonized with CHA0-*gfp2* strain or mock solvent were collected and their DNA used for real-time PCR using a 16S primer pair described in the STAR Methods. Ct values were normalized to Ct values obtained by a primer set (*AtACTIN2*) amplifying plant-derived DNA. Values are shown with means ± SD (3 biological replicates, see Figure S7E). Asterisks (^∗∗^*p* < 0.01 and ^∗∗∗^*p* < 0.001) indicate statistically significant differences based on ANOVA and Tukey’s test analysis. ns, not significant. (F) Schematic model of one of *PRRs*, *FLS2* expression pattern in *Arabidopsis* roots and damage-gated local MAMP responses during root-bacteria interaction. Plant roots request both presence of MAMPs and damage before mounting strong immune responses. This model can help to explain how these important *PRRs* can be usefully employed by plant roots, despite the continuous presence of high amounts of commensal or beneficial microbes while maintaining resistance to pathogenic, damage-inducing bacteria.

**Figure S7:**
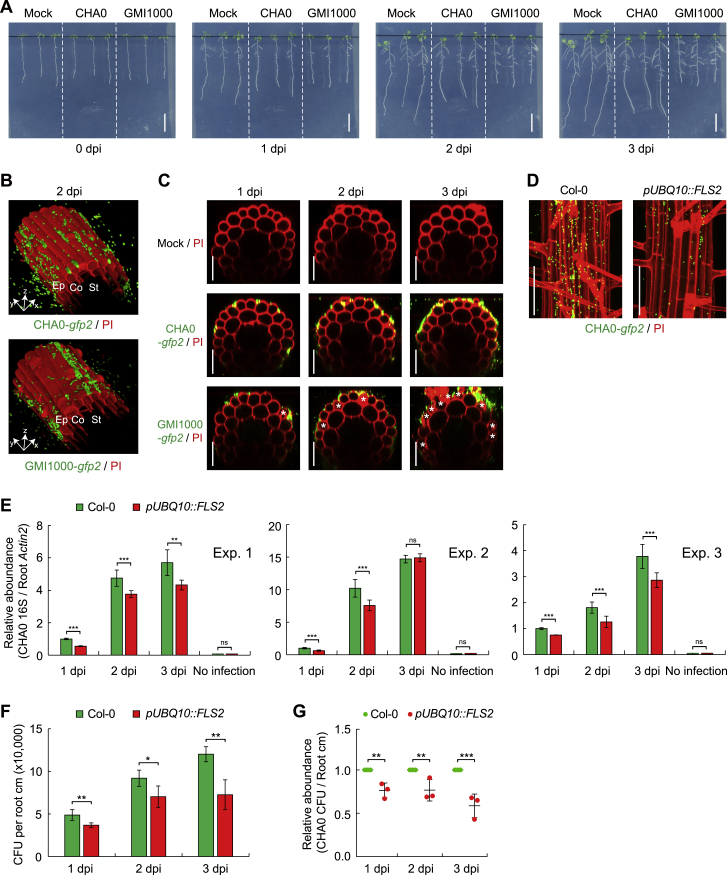
Bacterial Colonization of *Arabidopsis* Roots in an *In Vitro* System, Related to Figure 7 (A) Photographs of Col-0 roots infection with non-pathogenic (CHA0) or pathogenic (GMI1000) root bacteria on solid half MS medium plate. Six-day-old roots were inoculated with water (mock, left), CHA0 (middle) or GMI1000 (right) for the indicated time. Note pronounced root growth arrest in the presence of the pathogenic bacterium GMI1000. dpi, days post inoculation. Scale bar, 2 cm. (B) Bacterial colonization on the surface of differentiated epidermal cells in the view of the 3D-stacks. Pictures are maximum projections of confocal Z stacks taken around the 25th endodermal cell after onset of elongation. Ep, epidermis; Co, cortex; St, stele. (C) Orthogonal view of confocal images showing colonization and the extent of damage on epidermal cells after inoculation with CHA0 (middle panel) or GMI1000 (bottom panel) for the indicated time, compared to the mock (upper panel). White asterisks indicate damaged cells. Scale bar, 50 μm. (D) Representative images showing CHA0-*gfp2* colonization on differentiated roots of Col-0 and *pUBQ10::FLS2* root at 2 dpi. Pictures are maximum projections of confocal Z stacks. GFP-labeled bacteria (green) were co-visualized with PI staining (red). Scale bar, 50 μm. (E) Three biological replicates of quantitative measurement of CHA0 abundance in Col-0 and *pUBQ10::FLS2* roots at indicated inoculation time point. Roots inoculated with CHA0-*gfp2* strain or mock solvent were collected and their DNA was used for real-time PCR using CHA0 16S primer pair (499_500). Ct values were normalized to Ct values obtained by a primer pair (*AtACTIN2*) amplifying plant-derived DNA. Values are shown with means ± SD (n = 3 roots). (F and G) CFU counting of CHA0 colonization in Col-0 and *pUBQ10::FLS2* roots. Four-day-old seedlings were transferred onto half MS plates containing CHA0 (OD_600_ = 0.002). Three roots were collected for each sample at indicated colonization time point. CFU of CHA0 abundance was normalized to per root centimeter (cm) (F) and the ratio of bacterial abundance was relatively compared to Col-0 (G). Values are shown with means ± SD (3 biological replicates). Asterisks (^∗^*p* < 0.05, ^∗∗^*p* < 0.01 and ^∗∗∗^*p* < 0.001) indicate statistically significant differences based on ANOVA and Tukey’s test analysis.

Finally, we tested whether our observations are relevant in the context of actual, bacterial root colonization. For this, we first used the model commensal/beneficial *Pseudomonas protegens* strain CHA0 (CHA0) (Haas and Défago, 2005, Haas and Keel, 2003). Indeed, despite strong colonization of seedling roots on plates and floating hydroponic roots, no significant MAMP response could be observed in undamaged, differentiated roots (Figures 7A and S7A–S7C). However, when cell ablation was combined with colonization, the cells neighboring the damage site were showing a MAMP response to the presence of the bacteria (Figures 7B and 7C). As with flg22 treatments, MAMP responses to the bacteria were also observed around lateral root emergence sites and upon spontaneous damage (Figure 7A). Next, we tested a root pathogenic bacterium, *Ralstonia solanacearum* GMI1000 (GMI1000) (Genin and Boucher, 2004). Interestingly, GMI1000 colonization initially does not cause cell damage, nor a strong MAMP response (Figure 7D). However, progression of infection eventually leads to cell death of some epidermal cells, which is then associated with a localized upregulation of MAMP responses in neighboring cells (Figures 7D and S7A–S7C). Our bacterial colonization experiments demonstrate that cellular damage and lateral root emergence does not only unlock MAMP responsiveness to high doses of pure MAMPs such as flg22, or 3-OH-C10:0, but is also effective in unlocking responses to the more complex and probably much less concentrated cocktail of MAMPs associated with actual bacterial colonization. Interestingly, flg22 derived from GMI1000 flagellin was found not to activate the *Arabidopsis* FLS2 receptor (Pfund et al., 2004, Wei et al., 2018). This indicates that the damage-associated MAMP responses we observe upon GMI1000 infection must be caused by MAMPs other than flg22. In addition, the similar, local upregulation of MAMP responsiveness seen upon GMI1000-induced damage further suggests that the phenomenon we describe here is not specific to laser-ablation induced cell damage (already indicated by our observations that MAMP responsiveness also occurs adjacent to sites of spontaneous cell death).

Intriguingly, our constitutively expressing *pUBQ10::FLS2* line, showed direct MAMP responses to CHA0, in the absence of damage (Figure 7A). Such a constitutive, non-damage-gated defense activation should interfere with root colonization of a commensal bacterium such as CHA0 and might be quantifiable, in contrast to a local interference with microbial colonization upon laser-induced damage, which would be impossible for us to quantify. We indeed found a slight, but consistently lower degree of root colonization in plate assays in *pUBQ10::FLS2* lines, both by qPCR-based quantification and colony forming units (CFU) counting (Figures 7E and S7D–S7G). Thus, a restricted, damage-gated MAMP responsiveness of roots contributes to allow for root colonization by innocuous or beneficial bacterial species.

## Discussion

Plant roots generate an attractive environment for a subset of soil-borne microbes. These microbes, in turn, affect roots by manipulating plant hormones, signaling, nutrient acquisition, or growth of other microbes, using large sets of genes associated with their root-colonizing life-style (Levy et al., 2017). One important function that promotes colonization is thought to be the ability of some bacteria to suppress MAMP responses, thus avoiding production of anti-microbial compounds and inhibition of root growth. Suppression of MAMP perception by non-pathogenic colonizers has been reported, but is just starting to be understood in mechanistic terms (Garrido-Oter et al., 2018, Pel and Pieterse, 2013, Yu et al., 2019). Type III secretion system (T3SS) effectors are known to suppress MAMP perception (Chisholm et al., 2006), yet appear to be associated with a pathogenic (or symbiotic) life-style, with commensal/beneficial bacteria either not possessing a T3SS or containing only few recognizable T3SS proteins whose functions remain enigmatic (Loper et al., 2012, Stringlis et al., 2019). Our findings now provide an additional level of explanation of how non-pathogenic microbes can successfully colonize roots—by simply avoiding damage and the strong enhancement of immune responses that comes with it (Figure 7F). From the plant-side, such a damage-gating of immune responses is economical, as it avoids constitutive activation of defenses and localizes them to sites where aggressive microbial colonizers might induce cellular damage or where damage due to other causes has generated potential pathogen entry points. For innocuous, root-colonizing bacteria, such a system would alleviate the need to repress plant immunity, as long as colonization proceeds without damage. It will be intriguing to see whether the suppression of MAMP responses by non-pathogenic bacteria still allows for damage-induced enhancement of MAMP responsiveness, in contrast to suppression by type III effectors, which can directly interfere with signaling components downstream of MAMP receptors and can thus be expected to suppress MAMP perception in absence or presence of damage.

An initial pathogenic infection in soil is bound to be localized, involving one or a few cells. Manipulations and molecular readouts at single-cell resolution are therefore of crucial importance for a mechanistic understanding of root-microbe interactions. Recently, we reported that single-cell damage causes surface depolarization, actively propagating calcium signals, ROS, and ethylene production in a surprisingly large region around the single-cell wound (Marhavý et al., 2019). Here, we demonstrate that ablation of clusters of a few cells causes an ethylene-independent, much more restricted, upregulation of MAMP responsiveness, difficult, or impossible to observe by standard molecular readouts or standard methods of wounding. Recently, damage of root cap tissue in meristems was shown to lead to jasmonate receptor-dependent regeneration responses (Zhou et al., 2019). Although we have focused on the differentiated and transition/elongation zone of the root—in which we do not observe regeneration responses—it would be intriguing to investigate whether and how the damage-gating of immune responses described here can be integrated with tissue regeneration. A recent report proposes that loss of cellular integrity causes calcium increases, activating AtPEP1 processing and release into the apoplast, where it could report damage to neighboring cells (Hander et al., 2019). Yet, the damage-induced gain of MAMP responsiveness that we observe here is not reconstituted by co-treatment with AtPEP1 or other DAMPs. We therefore propose that local, non-propagating signals are additionally required for a damage response, such as mechanical stresses on neighboring cell walls or plasmodesmatal collapse, induced by loss of turgor and cellular disintegration in the neighbor. Our data suggest that DAMP release might be a necessary element of damage perception, but is, on its own, insufficient to reconstitute actual cellular damage. In the future, it will be fascinating to use single-cell damage to investigate the immediate molecular events and mechanism that translate loss of cellular integrity into immune responsiveness of adjacent cells.

## STAR★Methods

### Key Resources Table

**Table:** 

REAGENT or RESOURCE	SOURCE	IDENTIFIER
**Bacterial and Virus Strains**		

*Pseudomonas protegen*s CHA0	Voisard et al., 1989	NCBI:txid1124983
*Pseudomonas protegen*s CHA0-*gfp2*	Péchy-Tarr et al., 2013	N/A
*Ralstonia solanacearum* GMI1000	Granada and Sequeira, 1983	NCBI:txid267608
*Ralstonia solanacearum* GMI1000-*gfp2*	This paper	N/A

**Chemicals, Peptides, and Recombinant Proteins**		

flg22_CHA0_	Peptide Specialty Laboratories GmbH	N/A
AtPEP1	Peptide Specialty Laboratories GmbH	N/A
nlp20	Peptide Specialty Laboratories GmbH	N/A
elf18	Peptide Specialty Laboratories GmbH	N/A
TAMRA-flg22_Pa_	Peptron	N/A
TAMRA-AtPEP1	Peptron	N/A
Propidium iodide (PI)	Sigma-Aldrich	Cat#P4170
Extracellular ATP (eATP)	Sigma-Aldrich	Cat#A2383
D-(+)-Cellobiose	Sigma-Aldrich	Cat#C7252
(±)-3-Hydroxydecanoic acid (3-OH-C10:0)	Sigma-Aldrich	Cat#H3648
Chitin from shrimp shells	Sigma-Aldrich	Cat#C9752
Galacturonan oligosaccharide mixture DP10-DP15 (OGs)	Elicityl	GAT114
(±)-Abscisic acid (ABA)	Sigma-Aldrich	Cat#A1049
Aminoethoxyvinylglycine (AVG)	Sigma-Aldrich	Cat#A6685
1-Aminocyclopropane-1-carboxylic acid (ACC)	Sigma-Aldrich	Cat#A3903

**Critical Commercial Assays**		

MESA BLUE qPCR MasterMix Plus for SYBR Assay	Eurogentec	RT-SY2X-03+WOUB

**Experimental Models: Organisms/Strains**		

*Arabidopsis thaliana****:*** WT Col-0	NASC	NCBI:txid3702
*Arabidopsis*: *fls2*	Zipfel et al., 2004	SALK_062054C
*Arabidopsis: sgn3-3*	Pfister et al., 2014	SALK_043282
*Arabidopsis: esb1-1*	Hosmani et al., 2013	NASC ID: N2106042
*Arabidopsis: ein2-1*	Alonso et al., 1999	NASC ID: N65994
*Arabidopsis: etr1-1*	Chang et al., 1993	NASC ID: N237
*Arabidopsis: pGPAT5::mCITRINE-SYP122*	Barberon et al., 2016	Transgenic Col-0
*Arabidopsis: pPER5::NLS-3xmVENUS*	Poncini et al., 2017	Transgenic Col-0
*Arabidopsis: pPER5::NLS-3xmVENUS, pUBQ10::RCI2A-tdTomato*	This paper	Transgenic Col-0
*Arabidopsis: pWRKY11::NLS-3xmVENUS*	Poncini et al., 2017	Transgenic Col-0
*Arabidopsis: pWRKY11::NLS-3xmVENUS, pUBQ10::RCI2A-tdTomato*	This paper	Transgenic Col-0
*Arabidopsis: pMYB51::NLS-3xmVENUS*	Poncini et al., 2017	Transgenic Col-0
*Arabidopsis: pMYB51::NLS-3xmVENUS, pUBQ10::RCI2A-tdTomato*	This paper	Transgenic Col-0
*Arabidopsis: pFRK1::NLS-3xmVENUS*	This paper	Transgenic Col-0
*Arabidopsis: pFRK1::NLS-3xmVENUS, pUBQ10::RCI2A-tdTomato*	This paper	Transgenic Col-0
*Arabidopsis: pFLS2::NLS-3xmVENUS*	This paper	Transgenic Col-0
*Arabidopsis: pFLS2::NLS-3xmVENUS, pUBQ10::RCI2A-tdTomato*	This paper	Transgenic Col-0
*Arabidopsis: pFLS2*_*long*_*::NLS-3xmVENUS, pUBQ10::RCI2A-tdTomato*	This paper	Transgenic Col-0
*Arabidopsis: pEFR::NLS-3xmVENUS, pUBQ10::RCI2A-tdTomato*	This paper	Transgenic Col-0
*Arabidopsis: pCERK1::NLS-3xmVENUS, pUBQ10::RCI2A-tdTomato*	This paper	Transgenic Col-0
*Arabidopsis: pRLP23::NLS-3xmVENUS, pUBQ10::RCI2A-tdTomato*	This paper	Transgenic Col-0
*Arabidopsis: pLORE::NLS-3xmVENUS*	This paper	Transgenic Col-0
*Arabidopsis: pFLS2::FLS2-3xMYC-GFP*	Robatzek et al., 2006	Transgenic Ws-0
*Arabidopsis: pFLS2*_*long*_*::FLS2-3xMYC-mVENUS*	This paper	*fls2* mutant
*Arabidopsis: pPER5::NLS-3xmVENUS, pFLS2*_*long*_*::FLS2-3xMYC-mVENUS*	This paper	*fls2* mutant
*Arabidopsis: pUBQ10::FLS2*	This paper	Transgenic Col-0
*Arabidopsis: pFRK1::NLS-3xmVENUS, pUBQ10::FLS2*	This paper	Transgenic Col-0
*Arabidopsis: pPER5::NLS-3xmVENUS, pUBQ10::FLS2*	This paper	Transgenic Col-0
*Arabidopsis: pFRK1::NLS-3xmVENUS, pFLS2::NLS-tdTomato*	This paper	Transgenic Col-0
*Arabidopsis: pPER5::NLS-3xmVENUS, pFLS2::NLS-tdTomato*	This paper	Transgenic Col-0

**Oligonucleotides**		

Primers for cloning reporter lines, see Table S1	This paper	N/A
Primer: CHA0 16S gene Forward: TGAAGAAGGTCTTCGGAT TGTAAAGC	This paper	N/A
Primer: CHA0 16S gene Reverse: GCTACACAGGAAATTCCACCACCCT	This paper	N/A
Primer: *Arabidopsis* housekeeping gene *AtACTIN2* Forward: CTGGATCGGTGGTTCCATTC	This paper	N/A
Primer: *Arabidopsis* housekeeping gene *AtACTIN2* Reverse: CCTGGACCTGCCTCATCATAC	This paper	N/A

**Recombinant DNA**		

*pFRK1::NLS-3xmVENUS*	This study	N/A
*pPER5::NLS-3xmVENUS*	This study	N/A
*pWRKY11::NLS-3xmVENUS*	This study	N/A
*pMYB51::NLS-3xmVENUS*	This study	N/A
*pFLS2::NLS-3xmVENUS*	This study	N/A
*pFLS2*_*long*_*::NLS-3xmVENUS*	This study	N/A
*pEFR::NLS-3xmVENUS*	This study	N/A
*pCERK1::NLS-3xmVENUS*	This study	N/A
*pRLP23::NLS-3xmVENUS*	This study	N/A
*pLORE::NLS-3xmVENUS*	This study	N/A
*pFLS2::NLS-tdTomato*	This study	N/A
*pUBQ10::RCI2A-tdTomato*	This study	N/A
*pUBQ10::FLS2*	This study	N/A
*pFLS2*_*long*_*::FLS2-3xMYC-mVENUS*	This study	N/A

**Software and Algorithms**		

Fiji (ImageJ)	Schneider et al., 2012	https://imagej.nih.gov/ij/
Zeiss Zen 2011	https://www.zeiss.com/corporate/int/home.html	N/A
GraphPad Prism 7.0	https://www.graphpad.com	N/A

### Lead Contact and Materials Availability

Further information and requests for resources and reagents should be directed to and will be fulfilled by the Lead Contact, Niko Geldner (niko.geldner@unil.ch). Plasmids and transgenic plant seeds generated in this study will be made available on request, but we may require a payment and/or a completed Materials Transfer Agreement if there is potential for commercial application.

### Experimental Model and Subject Details

#### Plant material

*Arabidopsis thaliana* ecotype Columbia (Col-0) was used as wild-type control for all experiments. The *fls2* (SALK_062054C), and *sgn3-3* and *esb1-1* mutants were previously described (Zipfel et al., 2004, Pfister et al., 2014, Hosmani et al., 2013). The *ein2-1* and *etr1-1* mutants were provided by the Nottingham *Arabidopsis* Stock Centre (NASC) and was originally reported in Alonso et al. (1999) and Chang et al. (1993). MAMP response reporter lines *pPER5::NLS-3xmVENUS*, *pWRKY11::NLS-3xmVENUS* and *pMYB51::NLS-3xmVENUS* were described previously (Poncini et al., 2017). Suberization maker *pGPAT5::mCITRINE-SYP122* was generated and reported previously (Barberon et al., 2016). *pFLS2::FLS2-3xMYC-GFP* line was obtained from *Prof.* Thomas Boller’s group (Robatzek et al., 2006).

#### Plant growth conditions

For all experiments, plant seeds were surface-sterilized in 70% EtOH for 10 min, then washed twice in 99% ethanol and dried in sterile conditions. Seeds were stratified at 4°C in the dark on 0.8% half Murashige and Skoog (MS) agar plates without addition of sucrose. Plant roots were grown vertically for 6 d at 22°C under continuous days.

#### Bacterial strains and growth conditions

The GFP-tagged *Pseudomonas protegen*s strain, CHA0-*gfp2* (CHA0::attTn*7*-*gfp2*; Gm^r^) and the GFP-labeled *Ralstonia solanacearum* strain, GMI1000-*gfp2* (GMI1000::attTn*7*-*gfp2*; Gm^r^) were provided by *Prof.* Christoph Keel (Péchy-Tarr et al., 2013) and generated by electroporation transformation method (See in METHOD DETAILS), respectively. Bacterial strains were incubated overnight in liquid LB medium (1% tryptone, 0.5% yeast extract and 1% NaCl, for CHA0-*gfp2*) or BG medium (1% peptone, 0.1% Casamino acid, 0.1% yeast extract and 0.5% glucose, for GMI1000-*gfp2*) supplemented with 30 μl/ml gentamycin at 28°C. Bacterial cells were collected by centrifugation, and resuspended in sterile MiliQ water for further root inoculation assays.

### Method Details

#### Generation of transgenic lines

For generating expression constructs, the In-Fusion Advantage PCR Cloning Kit (Clontech), Gateway Cloning Technology (Invitrogen) and GreenGate Cloning System (Lampropoulos et al., 2013) were used. See Table S1 for primer details. All plasmids were transformed by heat shock into *Agrobacterium tumefaciens* GV3101 strain with or without pSoup plasmid and then transformed into the corresponding plant lines by floral dipping method (Clough and Bent, 1998, Zhang et al., 2006). Several independent transgenic lines were analyzed, and the strongest line of each construct was selected for further studies.

For labeling of the plasma membrane, *pUBQ10::RCI2A-tdTomato* construct was generated using a triple Gateway reaction recombining the following plasmids: pDONR P4-P1R-*pUBQ10*, pDONR 221-*RCI2A* (containing the coding sequence of the small plasma membrane localized protein RARE-COLD-INDUCIBLE 2A (AtRCI2A)), pDONR P2R-P3-*tdTomato* and pK7m34GW (destination vector containing the kanamycin resistance gene for *in planta* selection). The resulting plasmid was transformed into Col-0 plants. Transcriptional reporters were created using the following promoters: *pFRK1* (Asai et al., 2002), *pFLS2* (Zipfel et al., 2004), *pFLS2*_*long*_, *pEFR* (Zipfel et al., 2006), *pCERK1*(Miya et al., 2007), *pRLP23* (Albert et al., 2015), *pLORE* (Ranf et al., 2015). Fragments were PCR-amplified and cloned into HindШ site of *pGreenHygromycin-NLS-3xmVENUS* (Vermeer et al., 2014). The resulting constructs were introduced into Col-0 or *pUBQ10::RCI2A-tdTomato* background.

To overexpress *FLS2* gene in MAMP marker lines, the *pUBQ10::FLS2* plasmid was constructed using double Gateway cloning. The full-length genomic *FLS2* DNA, including the *FLS2* coding region, 227 bp of upstream sequence, and 953 bp downstream sequence was cloned into the entry clone pDONR 221. This vector was then combined to the entry clone pDONR P4-P1R-*pUBQ10* and the destination vector pK7m24GW to create the final expression clone *pUBQ10::FLS2*. The resulting construct was transformed into stable MAMP marker lines, which were then introduced into the *sgn3-3* mutant background by genetic crossing. For generating *FLS2* complementation line, the *pFLS2*_*long*_*::FLS2-3xMYC-mVENUS* plasmid was constructed by double Gateway cloning. Full-length genomic *FLS2* fragment fused with triple MYC tag followed by a mVENUS sequence was cloned into pDONR 221. This vector was then combined with an entry clone pDONR P4-P1R-*pFLS2*_*long*_ and the destination vector pFR7m24GW (destination vector containing the *FastRed* cassette for transgenic seed selection) (Shimada et al., 2010) to create the final expression clone, which was transformed into *fls2* mutant background.

To combine *FLS2* and MAMP-reporters in the same background, *pFLS2::NLS-tdTomato* plasmid was constructed using Greengate Cloning System. *pFLS2* short promoter was PCR-amplified and cloned into pGGA (plasmid Green Gate A) entry vector to generate pGGA-*pFLS2*, which was then recombined using Greengate reaction with the following plasmids: pGGB-*SV40-NLS*, pGGC*-tdTomato*, pGGD*-dummy*, pGGE*-UBQ10terminator*, pGGF*-FastRed* and pGGZ*-empty* destination vector. The final construct possesses the *FastRed* cassette for transgenic plant selection. The obtained construct was transformed into a stable MAMP marker background.

#### Elicitor, hormone and inhibitor treatments

flg22_CHA0_ oligopeptide from *Pseudomonas protegens* CHA0 (TRLSSGLKINSAKDDAAGLQIA) (Jousset et al., 2014), nlp20 oligopeptide from *Phytophthora parasitica* (*Pp*NLP) (AIMYSWYFPKDSPVTGLGHR) (Böhm et al., 2014), elf18 oligopeptide from *E. coli* strain GI826 (Ac-SKEKFERTKPHVNVGTIG) (Kunze et al., 2004) and *Arabidopsis thaliana* Plant Elicitor Peptide 1, AtPEP1 (ATKVKAKQRGKEKVSSGRPGQHN) (Yamaguchi et al., 2006) were chemically synthesized by Peptide Specialty Laboratories GmbH (https://www.peptid.de/). The peptides were dissolved in deionized water to obtain 1 mM stock solution and further dilutions were done with half MS medium. Fluorescently-labeled peptides TAMRA-flg22_Pa_ and TAMRA-AtPEP1 were synthesized by Peptron (http://www.peptron.com/) and dissolved in water to a final concentration of 1 μM for all assays. Extracellular ATP (eATP), D-(+)-cellobiose (cellobiose), (±)-3-Hydroxydecanoic acid (3-OH-C10:0) and chitin were obtained from Sigma-Aldrich. Galacturonan oligosaccharide mixture DP10-DP15 (OGs) was purchased from Elicityl (https://www.elicityl-oligotech.com/). These chemicals were dissolved in water to the stock concentrations of 100 mM for eATP, 1 mM for 3-OH-C10:0 and cellobiose, 2 mg/ml for chitin and 5 mg/ml for OGs. For hormone treatments, (±)-Abscisic acid (ABA) was stored as a 50 mM stock solution in methanol and 1-Aminocyclopropane-1-carboxylic acid (ACC) as a 20 mM stock solution in water. For ethylene biosynthesis inhibitor treatment, Aminoethoxyvinylglycine (AVG) was dissolved in water as a 10 mM stock solution.

For microscopic analysis of *pFLS2* reporter and MAMP marker lines under various treatments, six-day-old seedlings were carefully transferred into liquid half MS medium containing the mentioned chemical molecules using 12-well culture plates (CytoOne™). The seedlings were observed under confocal microscopy after 6h treatment, unless otherwise specified, in standard growth condition. A pool of 10-12 homozygous seedlings from the T3 generation was analyzed for each assay. At least three independent replicates were performed.

#### Confocal settings and image processing

Confocal laser scanning microscopy was performed on a Zeiss LSM880 inverted confocal scanning microscope. Pictures were taken with a 40 × water immersion objectives. For more detailed analyses in large area of interest, imaging was performed thanks to Z-scan with tile-scan (overlap 10%). For green and red fluorophores, the following excitation and detection windows were used: mVENUS/GFP 488 nm, 500-530 nm; mCITRINE 496 nm, 530 nm; PI 520 nm, 590 nm; tdTomato 550 nm, 580 nm; TAMRA 560 nm, 570-610 nm. Sequential scanning was used to avoid interference between fluorescence channels. Confocal images after treatments and/or ablations were taken following the “four identical criteria,” that is, using the same position in the roots, the same laser detection intensity, the same laser scanning area, and the same interval and number of slices for Z stack projection.

#### Laser ablation setup

The sample preparation and manipulation for laser ablation was done as described before (Marhavý et al., 2019). Briefly, six-day-old seedlings were carefully transferred from half MS medium plate into a Chambered Coverglass (Nunc Lab-Tek, 2-well format, Thermo Scientific). In each well 4-5 roots lied alongside the cover glass, and then the entire root parts were covered with a block of solid half MS medium (approximately equal to 1 mL in liquid volume). Finally, chambers were covered with lid and mounted onto the confocal microscopy for time-lapse imaging and cell-type-specific laser ablation. Cell ablation experiments were performed on a Zeiss LSM880 Confocal/Multiphoton (Mai-Tai Spectra-Physics Multiphoton laser). Parameters for ablation were set as below: 40 × water immersion objective, scaling dimensions (xyz), laser 800 nm −2%, beam splitter MBS_InVis: MBS 760+, pixel dwell: 0.8 μs. A region of interest (ROI) was drawn through the cell prior to ablation.

To combine laser ablation-caused cell damage with flg22 treatment in Chambered Coverglass system, we first ablated specific root cells and then immediately added 500 μL of 3 μM flg22 solution into the chamber to obtain a final concentration of 1 μM flg22. After 6h treatment, the liquid solution was removed carefully to avoid roots movement, and then confocal images were taken directly for reporter lines expressing the plasma membrane marker. For the lines devoid of plasma membrane marker, plasma membrane outline and damaged cells can be labeled clearly by adding 50 μL of PI solution (5x) onto the agar block of half MS medium for 10 min before observation.

#### Bacterial transformation and infection assay

To obtain the GFP-labeled *Ralstonia solanacearum* GMI1000 strain, GMI1000-*gfp2* (GMI1000::attTn*7*-*gfp2*; Gm^r^), we introduced a GFP fluorescent tag into the bacterial genome by electroporation transformation method as described before (Smith and Iglewski, 1989). Briefly, GMI1000 was grown in BG broth (1% Bacto peptone, 0.1% casamino acids, 0.1% yeast extract, 0.5% glucose) with vigorous shaking at 28°C until early log phase (OD_600_ = 0.4-0.6). 1.5 mL of pre-culture cells were harvested by centrifugation at 13,000 g for 2 min at 4°C, pellet was resuspended with the same volume MOPS-Glycerol (MOPS 1 mM with 15% Glycerol, keep on ice), re-centrifuged, washed in 1/3 volume of wash medium (MOPS-Glycerol) and finally re-suspended in 1/15 volume (75 μl) of MOPS-Glycerol. The cell suspension was chilled on ice for 30 min prior to electroporation. 5 μL of delivery vector, pBK-miniTn7-*gfp2* (Koch et al., 2001) and 5 μL of a helper plasmid DNA pUXBF13 (Bao et al., 1991), were gently mixed with cell suspension and then transferred to pre-chilled 0.2 cm cuvettes (Bio-Rad). Electroporation was performed using the following settings: capacitance, 25 μF; voltage, 2.4 kV; resistance, 200 Ω; pulse length, < 5 msec. 1 mL of SOC medium was then immediately added and the mix incubated with shaking for 1 h at 28°C. Finally, the mixture was plated on BG solid medium supplemented with 30 μl/ml gentamycin and incubated at 28°C until colonies have grown.

For bacterial infection on the roots, two different infection assays were used for both bacteria: drop dipping infection on solid MS plate and floating hydroponic inoculation. For drop dipping infection, we followed the method as described previously (Digonnet et al., 2012) with some modifications. In short, six-day-old seedlings were selected for uniform growth and transferred to half MS agar plates carefully. After incubation overnight in LB (for CHA0) or BG (for GMI1000) medium, bacteria were collected, washed and resuspended in distil water. 10 μL of bacterial suspension at an optical density of OD_600_ = 0.1 (10^8^ cfu/ml) was applied to the seedling by depositing small droplets along the whole root. Infected plates were then grown vertically for one to three days before microscopic observation according to the experiments. For floating hydroponic infection, four seeds were evenly spread on a small patch of sterile mesh (2 cm x 2 cm), which was then deposited onto a half MS agar plate for germination. After 3 days, when roots grew across the holes of mesh, we transferred the seedlings-supporting mesh onto a 12-well cell culture plates, containing 7 mL of hydroponic solution by well (the seedlings-supporting mesh floating on the solution). Grown for another 4 days, the bacterial suspension was then added in the hydroponic solution of each well to a final OD_600_ of 0.1. Roots were infected by bacteria for 6 h to 12 h before observation under confocal microscope.

For combining CHA0 infection with laser ablation, we used the Chambered Coverglass system similarly to flg22 treatment. Briefly, after ablation, 500 μL of bacterial suspension at an optical density of OD_600_ = 0.1 was gently added into the chamber to avoid roots movement. After 6 h infection, the bacterial solution was removed carefully, and confocal images were taken on Zeiss LSM 880.

#### Quantification of CHA0 colonization

For qPCR analysis of bacterial colonization, the experiment was performed as described previously (Garrido-Oter et al., 2018) with minor modification. In brief, four-day-old seedlings were carefully transferred to solid half MS plate containing CHA0 at final density of OD_600_ = 0.002. After inoculation at the indicated time point, three roots for each sample were collected from plates and briefly washed once in sterile water for 5 s to remove non-attached bacterial cells. After removal of excess water with a filter paper (Whatman, UK), roots were frozen in liquid nitrogen and stored at −80°C until further processing. DNA was extracted using the DNeasy Plant Mini Kit (QIAGEN, Germany) according to the manufacturer’s instructions. qPCR was performed in a 20 μL reaction mixture containing 10 μL MESA BLUE qPCR 2X MasterMix Plus for SYBR® Assay (Eurogentec, Belgium), 30 ng DNA template, 0.5 μM forward primer and 0.5 μM reverse primer. PCR was performed by a QuantStudio 3 Real-Time PCR System (Thermo Fisher Scientific, USA) using the following cycles: 95°C for 2 min, followed by 40 cycles of 95°C for 10 s, 58°C for 30 s, and 72°C for 30 s. Data from three biological replicates were analyzed following the delta-Ct method, which was used to estimate the relative abundance of bacteria to the abundance of plant DNA. Primers sequence used for qPCR are: 499_500 for CHA0 16S gene and plant housekeeping gene *AtACTIN2* for normalization.

For calculate the number of CHA0 colonization, the experiment was conducted by CFU counting (Saad et al., 2018). Briefly, four-day-old seedlings were transferred to new half MS agar plates containing CHA0 (OD_600_ = 0.002). Parts of their roots grown for indicated colonization time point were cut, gently washed by dipping in distilled water, and then ground in Eppendorf tubes using TissueLyser II (QIAGEN, Germany) with stainless steel beads. Each sample was resuspended in 500 μL of extraction buffer (10 mM MgCl_2_, 0.01% Silwet L-77) to homogenize the plant material. Samples were diluted 4,000-fold, and then spread on LB agar plates supplemented with 30 μl/ml gentamycin. The CFU were counted after 36h incubation at 28°C until colonies are clearly visible. Calculated number of CFU was normalized per centimeter of root length (total root length was determined based on images of root systems before their harvest). The experiment was conducted in three biological replicates, each with three technical replicates per condition; each sample consisted of three roots.

### Quantification and Statistical Analysis

For quantifying the nuclear-localized fluorescence intensity of MAMP markers and *FLS2* reporter, confocal images were analyzed with the Fiji package (http://fiji.sc/Fiji). Contrast and brightness were adjusted in the same manner for all images. In short, first, we set a defined threshold value for the same experiment between control and treatments. For example, all signals below a gray value threshold of 30 were excluded from quantification to avoid autofluorescence signal and weak non-MAMP responsive signal. Note that this threshold value is not fixed between different reporters and can be adjusted according to their fluorescent intensity. Second, after setting the detectable size of pixel to avoid noise signal, the size of the total area with signal (number of pixels) can be determined, which, multiplied by the average intensity of the pixels for each area, give the total fluorescence intensity for each nucleus, called “RawIntDen” - raw intensity density (RID). Finally, the overall score of an image is the sum of the RID values of all particles (nuclei).

Counting of the numbers of MAMP-responsive and/or *PRR*-positive cells in different root cell types was obtained as follows: a threshold value was set for removing noise signals. In some cases, for reporter lines or specific cell layers showing weak MAMP-responsive and/or *PRR*-positive fluorescence, we elevated the threshold value to separate the basal level of fluorescence and the weak non-MAMP responsive signals from the strongly induced MAMP-responsive signals. All signals below a given gray value threshold were excluded from the cell nuclei counting. The score average was obtained from 10-12 images of replicate roots.

All statistical analyses were done with the Graphpad Prism 7.0 software (https://www.graphpad.com/). One-way ANOVA was performed, and Tukey's test was subsequently used as a multiple comparison procedure. Details about the statistical approaches used can be found in the figure legends. The data are presented as mean ± SD, and “n” represents number of plant roots.

### Data and Code Availability

This study did not generate any unique datasets or code.

### Additional Resources

This study did not generate any additional resources.

## References

[bib1] Albert I., Böhm H., Albert M., Feiler C.E., Imkampe J., Wallmeroth N., Brancato C., Raaymakers T.M., Oome S., Zhang H. (2015). An RLP23-SOBIR1-BAK1 complex mediates NLP-triggered immunity. Nat. Plants.

[bib2] Alonso J.M., Hirayama T., Roman G., Nourizadeh S., Ecker J.R. (1999). EIN2, a bifunctional transducer of ethylene and stress responses in *Arabidopsis*. Science.

[bib3] Asai T., Tena G., Plotnikova J., Willmann M.R., Chiu W.-L., Gomez-Gomez L., Boller T., Ausubel F.M., Sheen J. (2002). MAP kinase signalling cascade in *Arabidopsis* innate immunity. Nature.

[bib4] Bao Y., Lies D.P., Fu H., Roberts G.P. (1991). An improved Tn7-based system for the single-copy insertion of cloned genes into chromosomes of gram-negative bacteria. Gene.

[bib5] Barberon M., Vermeer J.E.M., De Bellis D., Wang P., Naseer S., Andersen T.G., Humbel B.M., Nawrath C., Takano J., Salt D.E., Geldner N. (2016). Adaptation of Root Function by Nutrient-Induced Plasticity of Endodermal Differentiation. Cell.

[bib6] Beck M., Wyrsch I., Strutt J., Wimalasekera R., Webb A., Boller T., Robatzek S. (2014). Expression patterns of flagellin sensing 2 map to bacterial entry sites in plant shoots and roots. J. Exp. Bot..

[bib7] Böhm H., Albert I., Oome S., Raaymakers T.M., Van den Ackerveken G., Nürnberger T. (2014). A conserved peptide pattern from a widespread microbial virulence factor triggers pattern-induced immunity in *Arabidopsis*. PLoS Pathog..

[bib8] Boller T., Felix G. (2009). A renaissance of elicitors: perception of microbe-associated molecular patterns and danger signals by pattern-recognition receptors. Annu. Rev. Plant Biol..

[bib9] Boudsocq M., Willmann M.R., McCormack M., Lee H., Shan L., He P., Bush J., Cheng S.-H., Sheen J. (2010). Differential innate immune signalling via Ca(^2+^) sensor protein kinases. Nature.

[bib10] Boutrot F., Segonzac C., Chang K.N., Qiao H., Ecker J.R., Zipfel C., Rathjen J.P. (2010). Direct transcriptional control of the *Arabidopsis* immune receptor FLS2 by the ethylene-dependent transcription factors EIN3 and EIL1. Proc. Natl. Acad. Sci. USA.

[bib11] Castrillo G., Teixeira P.J.P.L., Paredes S.H., Law T.F., de Lorenzo L., Feltcher M.E., Finkel O.M., Breakfield N.W., Mieczkowski P., Jones C.D. (2017). Root microbiota drive direct integration of phosphate stress and immunity. Nature.

[bib12] Chang C., Kwok S.F., Bleecker A.B., Meyerowitz E.M. (1993). *Arabidopsis* ethylene-response gene ETR1: similarity of product to two-component regulators. Science.

[bib13] Chinchilla D., Bauer Z., Regenass M., Boller T., Felix G. (2006). The *Arabidopsis* receptor kinase FLS2 binds flg22 and determines the specificity of flagellin perception. Plant Cell.

[bib14] Chisholm S.T., Coaker G., Day B., Staskawicz B.J. (2006). Host-microbe interactions: shaping the evolution of the plant immune response. Cell.

[bib15] Clay N.K., Adio A.M., Denoux C., Jander G., Ausubel F.M. (2009). Glucosinolate metabolites required for an *Arabidopsis* innate immune response. Science.

[bib16] Clough S.J., Bent A.F. (1998). Floral dip: a simplified method for Agrobacterium-mediated transformation of *Arabidopsis thaliana*. Plant J..

[bib17] Digonnet C., Martinez Y., Denancé N., Chasseray M., Dabos P., Ranocha P., Marco Y., Jauneau A., Goffner D. (2012). Deciphering the route of *Ralstonia solanacearum* colonization in *Arabidopsis thaliana* roots during a compatible interaction: focus at the plant cell wall. Planta.

[bib18] Felix G., Duran J.D., Volko S., Boller T. (1999). Plants have a sensitive perception system for the most conserved domain of bacterial flagellin. Plant J..

[bib19] Finkel O.M., Castrillo G., Herrera Paredes S., Salas González I., Dangl J.L. (2017). Understanding and exploiting plant beneficial microbes. Curr. Opin. Plant Biol..

[bib20] Garrido-Oter R., Nakano R.T., Dombrowski N., Ma K.-W., McHardy A.C., Schulze-Lefert P., AgBiome Team (2018). Modular Traits of the Rhizobiales Root Microbiota and Their Evolutionary Relationship with Symbiotic Rhizobia. Cell Host Microbe.

[bib21] Geldner N. (2013). The endodermis. Annu. Rev. Plant Biol..

[bib22] Genin S., Boucher C. (2004). Lessons learned from the genome analysis of *ralstonia solanacearum*. Annu. Rev. Phytopathol..

[bib23] Gigolashvili T., Berger B., Mock H.-P., Müller C., Weisshaar B., Flügge U.-I. (2007). The transcription factor HIG1/MYB51 regulates indolic glucosinolate biosynthesis in *Arabidopsis thaliana*. Plant J..

[bib24] Gómez-Gómez L., Boller T. (2000). FLS2: an LRR receptor-like kinase involved in the perception of the bacterial elicitor flagellin in *Arabidopsis*. Mol. Cell.

[bib25] Gómez-Gómez L., Felix G., Boller T. (1999). A single locus determines sensitivity to bacterial flagellin in *Arabidopsis thaliana*. Plant J..

[bib77] Granada G.A., Sequeira L. (1983). Survival of *Pseudomonas solanacearum* in soil, rhizosphere, and plant roots. Can. J. Microbiol..

[bib26] Haas D., Défago G. (2005). Biological control of soil-borne pathogens by fluorescent pseudomonads. Nat. Rev. Microbiol..

[bib27] Haas D., Keel C. (2003). Regulation of antibiotic production in root-colonizing Peudomonas spp. and relevance for biological control of plant disease. Annu. Rev. Phytopathol..

[bib28] Hander T., Fernández-Fernández Á.D., Kumpf R.P., Willems P., Schatowitz H., Rombaut D., Staes A., Nolf J., Pottie R., Yao P. (2019). Damage on plants activates Ca2+-dependent metacaspases for release of immunomodulatory peptides. Science.

[bib29] Hosmani P.S., Kamiya T., Danku J., Naseer S., Geldner N., Guerinot M.L., Salt D.E. (2013). Dirigent domain-containing protein is part of the machinery required for formation of the lignin-based Casparian strip in the root. Proc. Natl. Acad. Sci. USA.

[bib30] Hruz T., Laule O., Szabo G., Wessendorp F., Bleuler S., Oertle L., Widmayer P., Gruissem W., Zimmermann P. (2008). Genevestigator v3: a reference expression database for the meta-analysis of transcriptomes. Adv. Bioinforma..

[bib31] Jacobs S., Zechmann B., Molitor A., Trujillo M., Petutschnig E., Lipka V., Kogel K.-H., Schäfer P. (2011). Broad-spectrum suppression of innate immunity is required for colonization of *Arabidopsis* roots by the fungus *Piriformospora indica*. Plant Physiol..

[bib32] Jeworutzki E., Roelfsema M.R.G., Anschütz U., Krol E., Elzenga J.T.M., Felix G., Boller T., Hedrich R., Becker D. (2010). Early signaling through the Arabidopsis pattern recognition receptors FLS2 and EFR involves Ca-associated opening of plasma membrane anion channels. Plant J..

[bib33] Jousset A., Schuldes J., Keel C., Maurhofer M., Daniel R., Scheu S., Thuermer A. (2014). Full-Genome Sequence of the Plant Growth-Promoting Bacterium *Pseudomonas protegens* CHA0. Genome Announc..

[bib34] Keinath N.F., Waadt R., Brugman R., Schroeder J.I., Grossmann G., Schumacher K., Krebs M. (2015). Live Cell Imaging with R-GECO1 Sheds Light on flg22- and Chitin-Induced Transient [Ca(2+)]cyt Patterns in *Arabidopsis*. Mol. Plant.

[bib35] Koch B., Jensen L.E., Nybroe O. (2001). A panel of Tn7-based vectors for insertion of the *gfp* marker gene or for delivery of cloned DNA into Gram-negative bacteria at a neutral chromosomal site. J. Microbiol. Methods.

[bib36] Kunze G., Zipfel C., Robatzek S., Niehaus K., Boller T., Felix G. (2004). The N terminus of bacterial elongation factor Tu elicits innate immunity in *Arabidopsis* plants. Plant Cell.

[bib37] Kutschera A., Dawid C., Gisch N., Schmid C., Raasch L., Gerster T., Schäffer M., Smakowska-Luzan E., Belkhadir Y., Vlot A.C. (2019). Bacterial medium-chain 3-hydroxy fatty acid metabolites trigger immunity in *Arabidopsis* plants. Science.

[bib38] Lacombe S., Rougon-Cardoso A., Sherwood E., Peeters N., Dahlbeck D., van Esse H.P., Smoker M., Rallapalli G., Thomma B.P.H.J., Staskawicz B. (2010). Interfamily transfer of a plant pattern-recognition receptor confers broad-spectrum bacterial resistance. Nat. Biotechnol..

[bib39] Lampropoulos A., Sutikovic Z., Wenzl C., Maegele I., Lohmann J.U., Forner J. (2013). GreenGate---a novel, versatile, and efficient cloning system for plant transgenesis. PLoS ONE.

[bib40] Levy A., Salas Gonzalez I., Mittelviefhaus M., Clingenpeel S., Herrera Paredes S., Miao J., Wang K., Devescovi G., Stillman K., Monteiro F. (2017). Genomic features of bacterial adaptation to plants. Nat. Genet..

[bib41] Loper J.E., Hassan K.A., Mavrodi D.V., Davis E.W., Lim C.K., Shaffer B.T., Elbourne L.D.H., Stockwell V.O., Hartney S.L., Breakwell K. (2012). Comparative genomics of plant-associated *Pseudomonas* spp.: insights into diversity and inheritance of traits involved in multitrophic interactions. PLoS Genet..

[bib42] Lotze M.T., Zeh H.J., Rubartelli A., Sparvero L.J., Amoscato A.A., Washburn N.R., Devera M.E., Liang X., Tör M., Billiar T. (2007). The grateful dead: damage-associated molecular pattern molecules and reduction/oxidation regulate immunity. Immunol. Rev..

[bib43] Macho A.P., Zipfel C. (2014). Plant PRRs and the activation of innate immune signaling. Mol. Cell.

[bib44] Marhavý P., Kurenda A., Siddique S., Dénervaud Tendon V., Zhou F., Holbein J., Hasan M.S., Grundler F.M., Farmer E.E., Geldner N. (2019). Single-cell damage elicits regional, nematode-restricting ethylene responses in roots. EMBO J..

[bib45] Mersmann S., Bourdais G., Rietz S., Robatzek S. (2010). Ethylene signaling regulates accumulation of the FLS2 receptor and is required for the oxidative burst contributing to plant immunity. Plant Physiol..

[bib46] Millet Y.A., Danna C.H., Clay N.K., Songnuan W., Simon M.D., Werck-Reichhart D., Ausubel F.M. (2010). Innate immune responses activated in *Arabidopsis* roots by microbe-associated molecular patterns. Plant Cell.

[bib47] Miya A., Albert P., Shinya T., Desaki Y., Ichimura K., Shirasu K., Narusaka Y., Kawakami N., Kaku H., Shibuya N. (2007). CERK1, a LysM receptor kinase, is essential for chitin elicitor signaling in *Arabidopsis*. Proc. Natl. Acad. Sci. USA.

[bib48] Naseer S., Lee Y., Lapierre C., Franke R., Nawrath C., Geldner N. (2012). Casparian strip diffusion barrier in *Arabidopsis* is made of a lignin polymer without suberin. Proc. Natl. Acad. Sci. USA.

[bib49] Navarro L., Zipfel C., Rowland O., Keller I., Robatzek S., Boller T., Jones J.D.G. (2004). The transcriptional innate immune response to flg22. Interplay and overlap with Avr gene-dependent defense responses and bacterial pathogenesis. Plant Physiol..

[bib50] Péchy-Tarr M., Borel N., Kupferschmied P., Turner V., Binggeli O., Radovanovic D., Maurhofer M., Keel C. (2013). Control and host-dependent activation of insect toxin expression in a root-associated biocontrol pseudomonad. Environ. Microbiol..

[bib51] Pel M.J.C., Pieterse C.M.J. (2013). Microbial recognition and evasion of host immunity. J. Exp. Bot..

[bib52] Pfister A., Barberon M., Alassimone J., Kalmbach L., Lee Y., Vermeer J.E., Yamazaki M., Li G., Maurel C., Takano J. (2014). A receptor-like kinase mutant with absent endodermal diffusion barrier displays selective nutrient homeostasis defects. eLife.

[bib53] Pfund C., Tans-Kersten J., Dunning F.M., Alonso J.M., Ecker J.R., Allen C., Bent A.F. (2004). Flagellin is not a major defense elicitor in Ralstonia solanacearum cells or extracts applied to *Arabidopsis thaliana*. Mol. Plant Microbe Interact..

[bib54] Poncini L., Wyrsch I., Dénervaud Tendon V., Vorley T., Boller T., Geldner N., Métraux J.-P., Lehmann S. (2017). In roots of *Arabidopsis thaliana*, the damage-associated molecular pattern AtPep1 is a stronger elicitor of immune signalling than flg22 or the chitin heptamer. PLoS ONE.

[bib55] Ranf S., Gisch N., Schäffer M., Illig T., Westphal L., Knirel Y.A., Sánchez-Carballo P.M., Zähringer U., Hückelhoven R., Lee J., Scheel D. (2015). A lectin S-domain receptor kinase mediates lipopolysaccharide sensing in *Arabidopsis thaliana*. Nat. Immunol..

[bib56] Robatzek S., Chinchilla D., Boller T. (2006). Ligand-induced endocytosis of the pattern recognition receptor FLS2 in *Arabidopsis*. Genes Dev..

[bib57] Roux S.J., Steinebrunner I. (2007). Extracellular ATP: an unexpected role as a signaler in plants. Trends Plant Sci..

[bib58] Saad M., de Zelicourt A., Rolli E., Synek L., Hirt H. (2018). Quantification of Root Colonizing Bacteria. Bio Protoc..

[bib76] Schneider C.A., Rasband W.S., Eliceiri K.W. (2012). NIH Image to ImageJ: 25 years of image analysis. Nat. Methods.

[bib59] Shimada T.L., Shimada T., Hara-Nishimura I. (2010). A rapid and non-destructive screenable marker, FAST, for identifying transformed seeds of *Arabidopsis thaliana*. Plant J..

[bib60] Smith A.W., Iglewski B.H. (1989). Transformation of *Pseudomonas aeruginosa* by electroporation. Nucleic Acids Res..

[bib61] Souza C.A., Li S., Lin A.Z., Boutrot F., Grossmann G., Zipfel C., Somerville S.C. (2017). Cellulose-Derived Oligomers Act as Damage-Associated Molecular Patterns and Trigger Defense-Like Responses. Plant Physiol..

[bib62] Stringlis I.A., Zamioudis C., Berendsen R.L., Bakker P.A.H.M., Pieterse C.M.J. (2019). Type III Secretion System of Beneficial Rhizobacteria *Pseudomonas simiae* WCS417 and *Pseudomonas defensor* WCS374. Front. Microbiol..

[bib63] Thor K., Peiter E. (2014). Cytosolic calcium signals elicited by the pathogen-associated molecular pattern flg22 in stomatal guard cells are of an oscillatory nature. New Phytol..

[bib64] Toyota M., Spencer D., Sawai-Toyota S., Jiaqi W., Zhang T., Koo A.J., Howe G.A., Gilroy S. (2018). Glutamate triggers long-distance, calcium-based plant defense signaling. Science.

[bib65] Vermeer J.E.M., von Wangenheim D., Barberon M., Lee Y., Stelzer E.H.K., Maizel A., Geldner N. (2014). A spatial accommodation by neighboring cells is required for organ initiation in *Arabidopsis*. Science.

[bib75] Voisard C., Keel C., Dèfago G. (1989). Cyanide production by *Pseudomonas fluorescens* helps suppress black root rot of tobacco under gnotobiotic conditions. EMBO J..

[bib66] Wang P., Calvo-Polanco M., Reyt G., Barberon M., Champeyroux C., Santoni V., Maurel C., Franke R.B., Ljung K., Novak O. (2019). Surveillance of cell wall diffusion barrier integrity modulates water and solute transport in plants. Sci. Rep..

[bib67] Wei Y., Caceres-Moreno C., Jimenez-Gongora T., Wang K., Sang Y., Lozano-Duran R., Macho A.P. (2018). The Ralstonia solanacearum csp22 peptide, but not flagellin-derived peptides, is perceived by plants from the Solanaceae family. Plant Biotechnol. J..

[bib68] Wyrsch I., Domínguez-Ferreras A., Geldner N., Boller T. (2015). Tissue-specific FLAGELLIN-SENSING 2 (FLS2) expression in roots restores immune responses in Arabidopsis fls2 mutants. New Phytol..

[bib69] Yamaguchi Y., Pearce G., Ryan C.A. (2006). The cell surface leucine-rich repeat receptor for AtPep1, an endogenous peptide elicitor in Arabidopsis, is functional in transgenic tobacco cells. Proc. Natl. Acad. Sci. USA.

[bib70] Yu K., Liu Y., Tichelaar R., Savant N., Lagendijk E., van Kuijk S.J.L., Stringlis I.A., van Dijken A.J.H., Pieterse C.M.J., Bakker P.A.H.M. (2019). Rhizosphere-Associated Pseudomonas Suppress Local Root Immune Responses by Gluconic Acid-Mediated Lowering of Environmental pH. Curr. Biol..

[bib71] Zhang X., Henriques R., Lin S.-S., Niu Q.-W., Chua N.-H. (2006). *Agrobacterium*-mediated transformation of *Arabidopsis thaliana* using the floral dip method. Nat. Protoc..

[bib72] Zhou W., Lozano-Torres J.L., Blilou I., Zhang X., Zhai Q., Smant G., Li C., Scheres B. (2019). A Jasmonate Signaling Network Activates Root Stem Cells and Promotes Regeneration. Cell.

[bib73] Zipfel C., Robatzek S., Navarro L., Oakeley E.J., Jones J.D.G., Felix G., Boller T. (2004). Bacterial disease resistance in Arabidopsis through flagellin perception. Nature.

[bib74] Zipfel C., Kunze G., Chinchilla D., Caniard A., Jones J.D.G., Boller T., Felix G. (2006). Perception of the bacterial PAMP EF-Tu by the receptor EFR restricts *Agrobacterium*-mediated transformation. Cell.

