# Supplementary material for: Co-incidence of Damage and Microbial Patterns Controls Localized Immune Responses in Roots
Source: Cell. 2020 Feb 6;180(3):440–453.e18. doi: 10.1016/j.cell.2020.01.013 (PMC7042715; doi:10.1016/j.cell.2020.01.013)
Supplement: Document S1. Table S1 [file mmc1.pdf]

Cell, Volume *180*

Supplemental Information

Co-incidence of Damage and Microbial Patterns Controls Localized Immune Responses in Roots

Feng Zhou, Aurélia Emonet, Valérie Dénervaud Tendon, Peter Marhavy, Dousheng Wu, Thomas Lahaye, and Niko Geldner

Table S1. Primers Used for Cloning Promoters of Different Reporter Lines in This Study, Related to STAR Methods.

Promoter line	AGI number	Forward primer	Reverse primer
pFRK1	At2g19190	ATCCCTGACAGTGAACTT	ATTGAGCTGCTTTCTCTG
pPER5	At1g14550	CTCAGTGCGTAGTAGTGAG	ATTTGTAGATCTCACTTGGT
pWRKY11	At4g31550	CTTCCCCACCCATATATAG	GATGATTTCTTGGTCTGAGGAT
pMYB51	At1g18570	TGTACTAAAGAACTACTGTAA	TCTTGATTCTTCAAACTTAGCT
pFLS2	At5g46330	TATTTTAAATCGCCTATTT	AAATCTATAGACGAAGTCA
pFLS2$_{long}$	At5g46330	CGTTTTATAGATGGATCGGGT	GGTTTAGACTTTAGAAGAGTTG
pEFR	At5g20480	TCCCTCACGTTCGATGGAG	CATTACAGTCGTGGGGCAAT
pCERK1	At3g21630	GCACTACAGGGCATGGACC	GCTTCCTTAGATTCCCCAGAG
pRLP23	AT2G32680	TGGTGGATTTGGTAAAGGCA	GGTTGTCTTGAAATATCTTTTA
pLORE	AT1G61380	ATCTGTGATTCAAGGGCGTTA	GGGGATAGAGTGAGCATTGAG
